# Hepatic safety of pretomanid- and pyrazinamide-containing regimens in TB Alliance clinical trials

**DOI:** 10.5588/ijtldopen.25.0199

**Published:** 2025-08-13

**Authors:** J. Nedelman, M. Li, M. Olugbosi, R. Bruning-Barry, J. Ambroso, M. Cevik, S. Gillespie, D.J. Sloan, M. Beumont, E. Sun

**Affiliations:** ^1^ TB Alliance, New York, USA;; ^2^ Current address, Merck & Co., Inc., Rahway, NJ, USA;; ^3^ RTI International, Research Triangle Park, USA;; ^4^ University of St. Andrews, St. Andrews, Scotland, UK.

**Keywords:** tuberculosis, hepatotoxicity, bedaquiline, linezolid, moxifloxacin

## Abstract

**BACKGROUND:**

In STAND and SimpliciTB, clinical trials for drug-susceptible TB, regimens containing pretomanid, pyrazinamide, and other agents (PaZX) had more hepatotoxicity than the standard-of-care regimen of isoniazid, rifampicin, pyrazinamide, and ethambutol (HRZE). In Nix-TB and ZeNix, clinical trials for drug-resistant TB, the regimen of bedaquiline, pretomanid, and linezolid (BPaL) demonstrated a favorable benefit-risk profile. We compare the hepatic safety of HRZE, PaZX, and BPaL in their respective populations.

**METHODS:**

In this post-hoc analysis of data from six clinical trials, rates of treatment-emergent elevations of alanine transaminase (ALT) during the first 8 weeks of treatment were estimated by Kaplan-Meier (KM) analysis and compared via log-rank testing and Cox modeling.

**RESULTS:**

The KM-estimated probabilities of treatment-emergent ALT elevations greater than 3x the upper limit of normal (>3xULN) were 5.36%, 12.7%, and 11.4% for HRZE, PaZX, and BPaL, respectively. The only significant (p < 0.05) difference was HRZE versus PaZX. The probabilities of ALT elevations >8xULN were 2.68%, 4.58%, and 1.05%, with the only significant difference being PaZX versus BPaL.

**CONCLUSIONS:**

BPaL and HRZE have similar hepatic safety profiles in their respective populations. Pretomanid and pyrazinamide should be co-administered only when the benefit outweighs the risk.

Pretomanid (Pa) is a nitroimidazole developed by TB Alliance. In 2019, the United States Food and Drug Administration approved Pa at 200 mg once daily as part of a 3-drug, 6-month regimen with bedaquiline (B) and linezolid (L) to treat adults with pulmonary, extensively drug-resistant TB (XDR-TB, pre-2021 definition) or intolerant or nonresponsive multidrug-resistant TB (MDR-TB):^1^ a regimen referred to as BPaL. In December 2022, the WHO recommended use of a 6-month regimen composed of BPaL with or without moxifloxacin (M) rather than the 9-month or longer regimens in patients with MDR-TB and rifampicin-resistant TB (RR-TB).^2^ Promising results from phase 2b studies of Pa combinations^3,4^ prompted exploration of treatment shortening. The STAND trial tested Pa with moxifloxacin and pyrazinamide (Z) PaMZ versus the standard of care HRZE (isoniazid, rifampin, pyrazinamide, and ethambutol) in drug-susceptible TB (DS-TB).^5^ STAND was paused following 3 deaths in the PaMZ arms associated with hepatotoxicity. After review of the safety data, the Safety Monitoring Committee recommended resuming enrollment, but TB Alliance instead pursued what appeared to be a more promising regimen, BPaMZ in the SimpliciTB trial.^6^ BPaMZ demonstrated more rapid sputum culture conversion but a higher rate of withdrawals due to elevated hepatic enzymes. After approval of Pa in the BPaL regimen, based on the Nix-TB study,^7^ TB Alliance conducted the ZeNix study,^8^ which further optimized the linezolid dose. In both studies with BPaL, there were no withdrawals due to elevated hepatic enzymes.

Hepatic reactions are the most common side effect of pyrazinamide.^9^ The HRZE regimen, in which H and R are also associated with hepatotoxicity,^10^ has served as the control arm in DS-TB in the TB Alliance studies. To understand the relative risks of different regimens used in different trials, in particular the roles of Pa and Z, we have conducted an exploratory, retrospective analysis of individual-patient data from all the studies conducted by TB Alliance of at least 8 weeks duration. The primary objective of the analysis was to compare regimens containing Pa and Z (PaZX), BPaL, and HRZE through 8 weeks of treatment in their respective populations. Other aspects of hepatic safety (potential Hy’s Law cases) were also reviewed.

## METHODS

Data were pooled from all six completed studies conducted by TB Alliance of Pa in participants with pulmonary TB where the treatment duration was at least eight weeks: NC-002^3^ (8 weeks; PaMZ versus HRZE in DS-TB, PaMZ in MDR-TB), NC-005^4^ (8 weeks; BPaZ versus HRZE in DS-TB, BPaMZ in MDR-TB), STAND^5^ (4 and 6 months PaMZ versus 6 months HRZE in DS-TB, 6 months PaMZ in MDR-TB), Nix-TB^7^ (6 months; BPaL in highly resistant TB), ZeNix^8^ (6 months; BPaL in highly resistant TB), and SimpliciTB^6^ (4 months BPaMZ versus 6 months HRZE in DS-TB; 6 months BPaMZ in MDR-TB). All research protocols were approved by institutional review boards or ethics committees, and all participants gave written informed consent. More details about the studies are provided in the Supplementary Data.

Participants included in the analysis were grouped into three sets of regimens: 1) HRZE, 2) PaZ-containing regimens (PaZX), and 3) BPaL. These regimen groups were compared based on treatment-emergent elevations of alanine aminotransferase (ALT), which was selected as an indicator of liver injury because it is considered more specific than aspartate aminotransferase (AST) as well as more objective than adverse event reports.^11^ In the PaZX regimens, Pa was dosed as 100 mg or 200 mg once daily (QD), M as 400 mg QD, and Z as 1500 mg QD. Bedaquiline was dosed as 400 mg QD for 2 weeks followed by 200 mg thrice weekly in one BPaZ arm of NC-005 and in Nix-TB, or as 200 mg QD for 8 weeks in one BPaZ arm and the BPaMZ arm of NC-005, or as 200 mg QD for 8 weeks followed by 100 mg QD in SimpliciTB and ZeNix. In BPaL, Pa was dosed as 200 mg QD. Linezolid was dosed starting at 1200 mg QD in Nix-TB and starting at 600 mg QD or 1200 mg QD in ZeNix; the duration was 26 weeks in Nix-TB and 9 weeks or 26 weeks in ZeNix; in both studies, L dose adjustments for toxicity were allowed. HRZE was dosed by weight bands according to standard practice.^12^

The six studies were conducted at different stages of Pa development, spanned a decade, and had different schedules of laboratory safety assessments (Supplementary Data Table S1); and medical monitoring activities evolved over time. Later protocols had more specific hepatic-safety guidelines, but investigators still had discretion to perform unscheduled visits and to manage participants. Details of hepatotoxicity monitoring guidelines for all studies are provided in the Supplementary Data. All 6 studies used the DMID toxicity scale (2007 draft^13^; see Supplementary Data), where ALT elevations between >3x and 8x the upper limit of normal (>3xULN to 8xULN) were classified as grade 3, and ALT elevations more than 8xULN were classified as grade 4. Therefore, elevations >3xULN and >8xULN were the primary focus here, but elevations >5xULN and >10xULN were also examined in alignment with FDA guidance.^11^

All participants who received at least one dose of the relevant study regimens, had at least one post-dose ALT measurement, and did not have a baseline value of ALT exceeding the threshold elevation level (3xULN, 5xULN, 8xULN, or 10xULN) were included in the analysis for treatment-emergent exceedance of the given threshold elevation level. Two studies had only 8 weeks of treatment, and in the other 4 most first occurrences of ALT elevation were by 8 weeks. So, our focus was on the first 8 weeks of treatment. For probability calculations, the 8-week timepoint was taken to be Day 60. Other methodological details regarding temporal attribution of events are provided in the Supplementary Data.

### Primary analysis

The three regimen groups’ probabilities of treatment-emergent ALT elevations during the first 8 weeks of treatment were estimated using Kaplan-Meier analysis of time to first occurrence that censored participants who withdrew before the end of 8 weeks or who did not have an elevation by 8 weeks. Also, the probability of an elevation of >3xULN progressing to an elevation >8xULN was estimated by restricting the Kaplan-Meier analysis of the latter event to participants experiencing the former event (and similarly for the progression from >3xULN to >10xULN). All estimated probabilities were compared across the three regimens using log-rank tests.

### Secondary analyses

Two types of secondary analyses were undertaken: 1) Cox regression modelling; 2) qualitative review of other aspects of hepatic safety.

Three sets of Cox regression models were applied to assess whether differences among regimen groups identified in the primary analysis remained after accounting for participant characteristics that may vary in distribution across the non-randomized groups. In the first set, the same three regimen groups of HRZE, PaZX, and BPaL were compared accounting for main effects of baseline xULN, age, weight, sex, race, and HIV status. The second and third sets assessed the influence of pretomanid pharmacokinetic exposure and DS-TB versus DR-TB. In the second set, only the pretomanid-containing regimens PaZX and BPaL were compared. To adjust for different pretomanid doses and possible pharmacokinetic interactions, steady-state pretomanid exposure, quantified by the 24-hour area under the curve of pretomanid concentration (AUC_0-24_), available from a published population pharmacokinetic model^14^ and unpublished extensions thereof, was also included as a covariate. Additionally, the PaZX group was subdivided into participants with DS-TB (PaZX-DS) or DR-TB (PaZX-DR). All participants on BPaL had DR-TB. In the third set of models, PaZX-DS and HRZE were compared accounting for main effects of baseline xULN, age, weight, sex, race, and HIV status. HRZE is the standard of care for, and is only used in, DS-TB. All three sets contained models for ALT elevations >3xULN and >8xULN. Hypotheses of proportional hazards were tested via the R cox.zph function^15^, and remediations guided by Schoenfeld residuals^15^ were applied when significant departures were detected.

Treatment-emergent potential Hy’s Law^11^ cases, defined as participants with ALT or AST >3xULN, total bilirubin >2xULN, and alkaline phosphatase (ALP) <2xULN, were enumerated. Narratives were provided for deaths in the PaZX group attributed to adverse liver events.

### Stastical significance

For all hypothesis tests (log-rank tests, Cox-model inferences), the threshold of p < 0.05 was used to judge ‘statistical significance’, even though all analyses were exploratory and post hoc, and no adjustments were made for multiplicity.

## RESULTS

**Figure 1.**
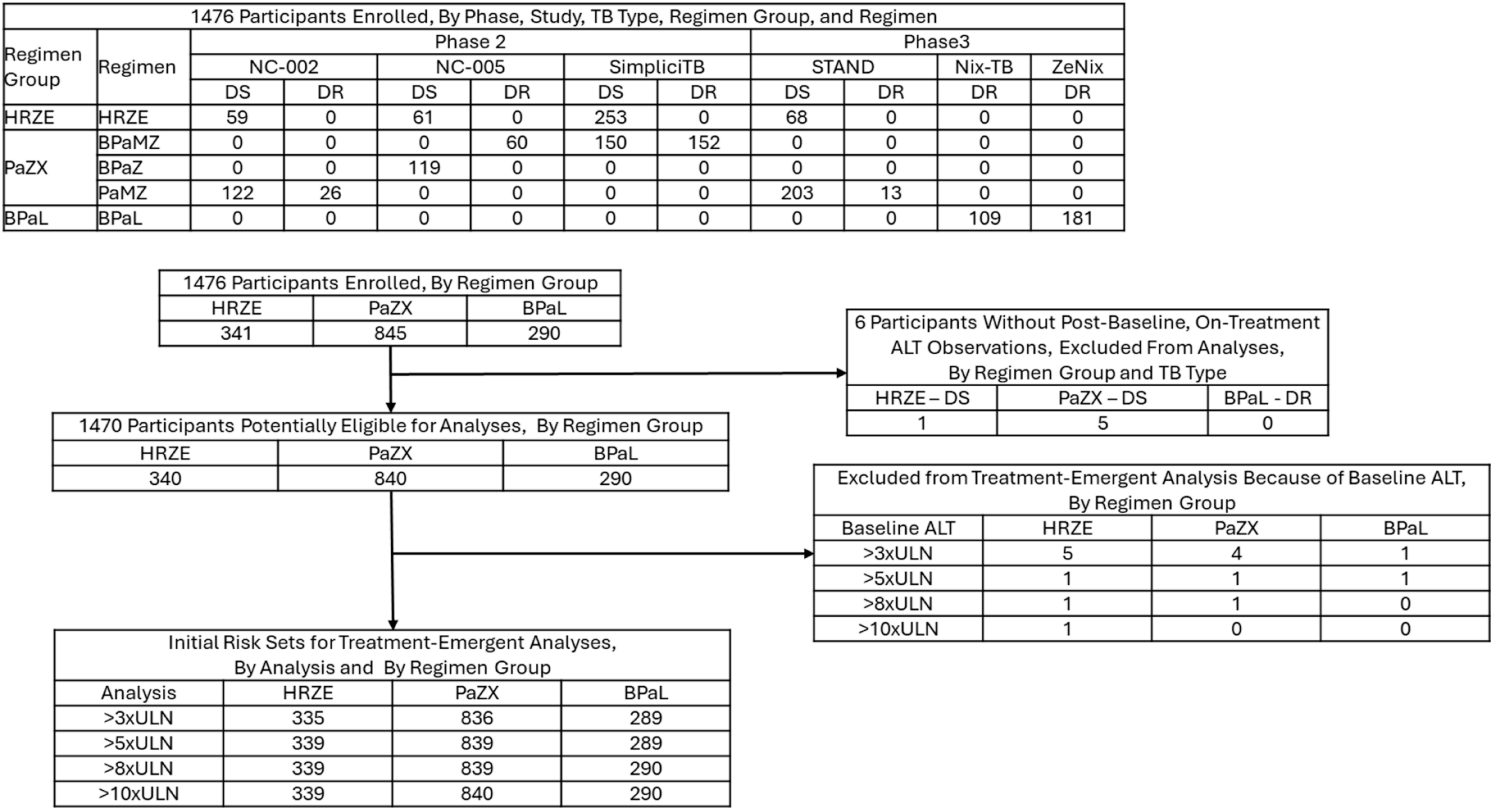
Participant flow from enrollment through risk sets. B = Bedaquiline; E = Ethambutol; H = Isoniazid; L = Linezolid; M = Moxifloxacin; Pa = Pretomanid; R = Rifampicin; X = Other drug(s); Z = Pyrazinamide; DR = drug resistant; DS = drug susceptible; ALT = alanine transaminase; ULN = upper limit of normal.

The flow of participants from enrollment through inclusion in the analyses based on the four ALT elevation thresholds is shown in Figure 1. Supplementary Data Table S2 provides additional information about participant flow through outcomes, and Table 1 summarizes patient characteristics. Participants on BPaL were more frequently white or living with HIV. By design, all participants on BPaL had DR-TB and all on HRZE had DS-TB; 70.5% of participants in the PaZX group had DS-TB.

**Table 1.** Table of summary statistics of covariates.

Variable	PaZX	BPaL	HRZE
n	841	290	340
Baseline xULN: Mean (SD) [N>3xULN; N>8xULN]	0.593 (0.529) [4; 1]	0.664 (0.555) [1; 0]	0.611 (0.801) [5; 1]
Age (years): Mean (SD)	35.1 (12.1)	36.6 (10.4)	34.8 (11.5)
Weight (kg): Mean (SD)	55.4 (11.1)	60.8 (13.7)	55.0 (10.4)
Pretomanid AUC_0-24_ (mg.h/L): Mean (SD)	73.3 (31.0)^1^	71.6 (31.5)^2^	NA
Sex Female: N (%)	259 (30.8)	111 (38.3)	88 (25.9)
Race Asian: N (%)	36 (4.3)	0 (0.0)	7 (2.1)
Race Black: N (%)	586 (69.7)	149 (51.4)	254 (74.7)
Race Other: N (%)	147 (17.5)	25 (8.6)	51 (15.0)
Race White: N (%)	72 (8.6)	116 (40.0)	28 (8.2)
HIV Positive: N (%)	185 (22.0)	92 (31.7)	67 (19.7)
TB Type DS: N (%)	593 (70.5)	0 (0.0)	340 (100)

B = bedaquiline; E = ethambutol; H: soniazid; L: Linezolid; Pa: Pretomanid; R = Rifampicin; X = Other drug(s); Z = Pyrazinamide; ULN = upper limit of normal; SD = standard deviation; N = Number kg = kilograms; mg = milligram; h = hours; L = litre; DS = drug susceptible.

1 n=831.

2 n=277.

### Primary analysis

**Figure 2.**
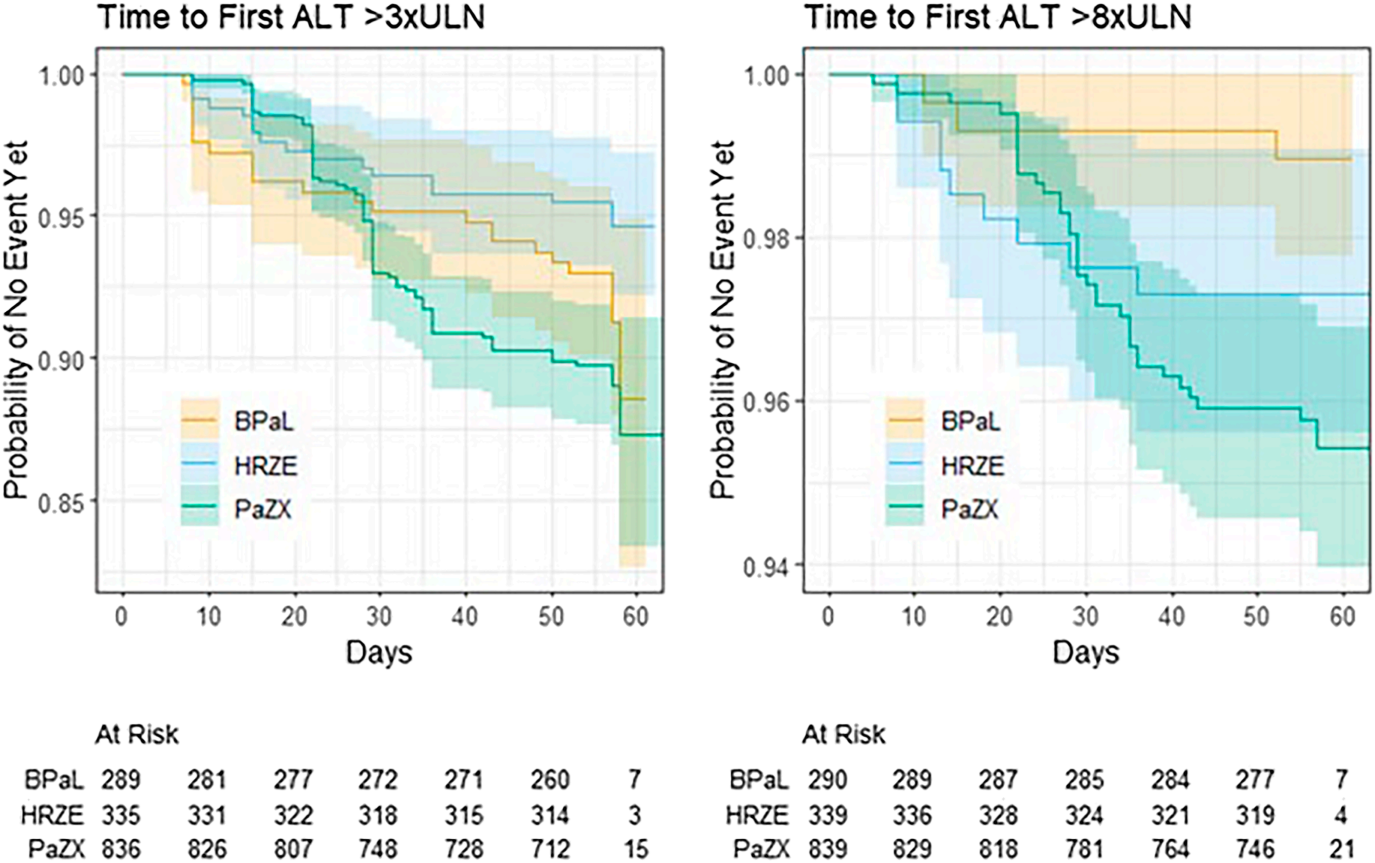
Time to first elevation of more than 3xULN and More than 8xULN by regimen group. B = Bedaquiline; E = Ethambutol; H = Isoniazid; L = Linezolid; M = Moxifloxacin; Pa = Pretomanid; R = Rifampicin; X = Other drug(s); Z = Pyrazinamide; DR = drug resistant; DS = drug susceptible; ALT = alanine transaminase; ULN = upper limit of normal.

Table 2, Figure 2 and Supplementary Data Figure S1 summarize the primary analysis. PaZX had a significantly greater risk than HRZE for ALT elevations >3xULN, and PaZX had a significantly greater risk than BPaL for ALT elevations >8xULN. For those who developed an elevation >3xULN, the probability of progressing to >8xULN was significantly lower for BPaL versus either HRZE or PaZX. Figure 2 shows qualitatively different profiles of time to first elevation, most evident for >8xULN, with the PaZX curve relatively flat through three weeks but then accelerating and crossing the curves for BPaL and HRZE.

**Table 2.** Probabilities of treatment-emergent ALT elevation during the first 8 weeks of treatment by different pooling groups.

Statistic	HRZE	PaZX	BPaL	p-values^**A**^
P(>3xULN)^**B**^	17/335 5.36 [2.82–7.82]%	88/836 12.7 [8.59–16.6]%	25/289 11.4 [5.07–17.3]%	0.011 0.003, 0.098, 0.276
P(>5xULN)	13/339 3.87 [1.79–5.92]%	61/839 7.71 [5.82–9.55]%	9/289 3.34 [1.16–5.47]%	0.006 0.027, 0.596, 0.009
P(>8xULN)	9/339 2.68 [0.939–4.40]%	36/839 4.58 [3.10–6.03]%	3/290 1.05 [0–2.23]%	0.018 0.177, 0.136, 0.007
P(>10xULN)	4/339 1.20 [0.0240–2.36]%	36/840 4.57 [3.09–6.02]%	2/290 0.692 [0–1.64]%	0.001 0.007, 0.523, 0.003
P(>8xULN|>3xULN)^**C**^	8/17 50.9 [18.0–70.6]%	35/88 45.0 [32.1–55.4]%	3/25 12.0 [0–23.9]%	0.006 0.16, 0.005, 0.007
P(>10xULN|>3xULN)	4/17 28.7 [0–49.5]%	34/88 43.9 [31.0–54.4]%	2/25 8.00 [0–18.0]%	0.016 0.639, 0.122, 0.004

A p-values are for tests of equal probabilities within a row. The top p-value is for the test of all three probabilities being equal, HRZE=PaZX=BPaL. The bottom three p-values are for HRZE=PaZX, HRZE=BPaL, PaZX=BPaL, respectively.

B P(>nxULN) = (Number of Participants with Events)/(Number Without ALT > nxULN at Baseline); Estimated probability of Treatment-Emergent ALT >nxULN by Day 60 [95% Confidence Interval], computed by Kaplan-Meier analysis.

C P(>nxULN|>3xULN) = (Number of Participants with Events)/(Number With Treatment Emergent ALT > 3xULN); Estimated Probability of Treatment-Emergent ALT > nxULN by Day 60, Given Treatment-Emergent >3xULN by Day 60, computed by Kaplan-Meier analysis restricted to participants with Treatment-Emergent ALT >3xULN.

B = Bedaquiline; E = Ethambutol; H = Isoniazid; L = Linezolid; Pa = Pretomanid; R = Rifampicin; X = Other drug(s); Z = Pyrazinamide; ULN = Upper Limit of Normal.

### Secondary analyses: Cox modeling

Table 3 summarizes results for >3xULN. As in the primary analysis, PaZX was found to have significantly greater risk than HRZE. In addition, here PaZX was found to have significantly greater risk than BPaL. HRZE and BPaL were not significantly different from each other. Increasing baseline ALT, age, and weight were all associated with increasing risk, as were female, non-Black, and HIV-positive status. Supplementary Data Table S3 shows similar results for >8xULN. PaZX was the reference group, with main effects for HRZE and BPaL. As in the primary analysis, PaZX had significantly greater risk than BPaL. The hypothesis of proportional hazards was rejected for HRZE. Based on Schoenfeld residuals, a piecewise-linear effect was modeled, decreasing to 40 days then flat. The hazard rate was significantly greater than that of PaZX initially but significantly smaller at Day 40. The hazard rate for HRZE was also significantly greater than that of BPaL initially but not later. Covariates had qualitatively similar effects as with >3xULN, but weight and HIV status were not statistically significant. Baseline also required a time-dependent hazard, decreasing piecewise linearly to Day 25.

**Table 3.** Results from Cox model for time to first elevation more than 3xULN, HRZE versus PaZX versus BPaL.

Variable	Coefficient	Hazard Ratio	Standard Error of Coefficient	p-value
HRZE:PaZX	-0.688	0.502	0.266	0.010
BPaL:PaZX	-0.568	0.567	0.235	0.016
HRZE:BPaL	-0.121	0.886	0.322	0.708
Baseline	0.650	1.92	0.171	< 0.001
Age	0.0177	1.02	0.00752	0.019
Weight	0.0148	1.01	0.00658	0.024
Female	0.421	1.52	0.189	0.026
Non-Black	0.491	1.63	0.206	0.017
HIV Positive	0.453	1.57	0.221	0.041

From a Cox model with reference values PaZX, Baseline xULN = 0.6, Age = 35 years, Weight = 55 kg, Male, Black, HIV negative. P-values based on standard normal distribution for Coefficient/Standard Error of Coefficient. Inference for HRZE:BPaL derived from estimates and covariance matrix for HRZE:PaZX and BPaL:PaZX.

B = Bedaquiline; E = Ethambutol; H = Isoniazid; L = Linezolid; Pa = Pretomanid; R = Rifampicin; X = Other drug(s); Z = Pyrazinamide

Supplementary Data Tables S4 and S5 show results comparing PaZX-DS, PaZX-DR, and BPaL, with pretomanid AUC as an additional covariate. The hazard rate for PaZX-DS was significantly greater than that of both PaZX-DR and BPaL, and the latter two were not significantly different. Pretomanid AUC did not have a significant effect. Supplementary Data Tables S6 and S7 show results comparing HRZE versus PaZX-DS. For >3xULN, HRZE had a significantly lower hazard rate than PaZX-DS. For >8xULN, the hypothesis of proportional hazards was rejected, and HRZE was found to have a significantly higher hazard rate initially and a significantly lower hazard rate later.

### Other aspects of hepatic safety

There were 3, 7, and 2 treatment-emergent potential Hy’s Law cases in the HRZE, PaZX, and BPaL groups, respectively. The two cases from BPaL had clear alternative etiology. See Supplementary Data Table S8 for details.

### Deaths

Narratives of deaths in the PaZX group attributed to adverse liver events are presented in the Supplementary Data.

## DISCUSSION

In this analysis across 6 studies with almost 1,500 participants, we characterized the hepatic safety profiles of three treatment regimens: HRZE, PaZX, and BPaL. ALT was selected as the indicator of liver injury because of its specificity, and compared to PaZX, the BPaL group had lower rates of ALT elevations >8xULN. However, Hy’s Law cases, defined also in terms of bilirubin, AST, and ALP, were reviewed. Rates were low across regimen groups, and the two cases from BPaL had clear alternative etiology. Hepatotoxicity is an established risk of pyrazinamide,^16–19^ so the behavior of PaZX may be due to Z. However, ALT elevations were found to be more frequent for PaZX than for HRZE, albeit with HRZE having higher hazard early in treatment, suggesting a contribution of Pa or interaction between Pa and Z. The observation of greater risk for PaZX-DS versus PaZX-DR and the excess hazard for PaZX relative to HRZE later in treatment suggests an immunological mechanism may be involved in the process. For the first time, BPaL is currently being tested in a DS-TB population in the NC-009 study (NCT06058299). This will provide an opportunity to determine whether the difference between DS-TB and DR-TB for PaZX applies also to BPaL.

The preclinical program of Pa found no evidence of hepatotoxic potential at clinical exposure levels. The potential for Pa to cause liver changes was determined in repeat-dose toxicity studies in rodents and monkeys up to 39 weeks duration.^20^ Although hepatocellular hypertrophy was seen in mice, rats, and monkeys given daily oral doses of Pa at dose levels ≥100 mg/kg/day (which is ≥487 mg human-equivalent dose assuming a 60 kg patient^21^), it was considered an adaptive response associated with increased metabolism. The Pa plasma exposures at doses where no adverse liver changes occurred ranged from approximately 2- to 5-fold higher than the clinical efficacious exposure. Pa monotherapy for up to 2 weeks was well tolerated in healthy participants^22–24^ and participants with TB.^25,26^ Healthy participants received Pa 50–1500 mg single dose and 200–1000 mg multiple doses for 7 or 8 days. One healthy participant (1/289, 0.35%) on Pa multiple dose 200 mg had a treatment-emergent ALT elevation that was between 3xULN and 8xULN. Participants with DS-TB received 50–1200 mg Pa for 14 days in two early bactericidal activity studies. One participant (1/122, 0.82%) on Pa multiple dose 1200 mg had a treatment-emergent ALT elevation between 3xULN and 8xULN.

Distributions of race, HIV status, and TB type varied across studies. Cox regression analysis was used to assess the effects of and control for these and other baseline characteristics. Results corroborated the higher risk of PaZX.

This exploratory, retrospective analysis has several limitations. Samples sizes were small relative to the rarity of the events of interest, reducing precision and power. There were imbalances in participant characteristics, exposures, and pyrazinamide doses between regimens in the cross-study comparisons. The studies were conducted over the span of a decade, and changes in patient care for TB, HIV and other comorbidities could have influenced observations. Study designs and medical monitoring activities have also evolved with time and experience. Although guidelines were provided for handling hepatoxicity, investigators could decide how to manage participants, potentially introducing additional heterogeneity. Moreover, the studies were open-label or partially so, which could have influenced investigators’ decisions. Geography might be another important factor, as different countries may have different practices; some of the trials were conducted only in one or a few countries while other trials had broader footprints. Other limitations include different frequencies of laboratory testing in the first 8 weeks, and incomplete information to systematically assess concurrent risk factors of liver injury such as co-infection with viral hepatitis, concomitant medications, alcohol use, and malnutrition.

## CONCLUSION

ALT elevations that occurred in different Pa-containing regimens had different characteristics. Participants receiving PaZ-containing regimens had a higher incidence of elevations >8xULN compared with participants receiving BPaL. The hepatic safety profile of BPaL in its indicated DR-TB population has been generally similar to that of HRZE in its indicated DS-TB population. We recommend that Pa and Z should only be co-administered when the benefit outweighs the risk.

## Supplementary Material


