# Supplementary material for: Hepatic safety of pretomanid- and pyrazinamide-containing regimens in TB Alliance clinical trials
Source: IJTLD Open. 2025 Aug 13;2(8):464–70. doi: 10.5588/ijtldopen.25.0199 (PMC12352949; doi:10.5588/ijtldopen.25.0199)
Supplement: Supplementary file 1 [file ijtldopen25-0199_supplementarydata1.pdf]

SUPPLEMENTARY MATERIAL

Hepatic Safety Profile of Pretomanid With and Without Pyrazinamide

Jerry Nedelman[1], Mengchun Li[1*] Morounfolu Olugbosi[1], Rebecca Bruning-Barry[2], Jeffrey Ambroso[2], Muge Cevik[3], Stephen Gillespie[3], Derek J Sloan[3], Maria Beumont[1], Eugene Sun[1]

[1]TB Alliance
[*] currently at Merck & Co., Inc.
[2]RTI International
[3]University of St. Andrews

Descriptions of studies

NC-002

Title: A Phase 2 Open-Label Partially Randomized Trial to Evaluate the Efficacy, Safety, and Tolerability of the Combination of Moxifloxacin plus PA-824 plus Pyrazinamide after 8 weeks of Treatment in Adult Patients with Newly Diagnosed Drug-Sensitive or Multidrug-Resistant, Smear-Positive Pulmonary Tuberculosis[1]

Dates: First-patient-first-visit 20 March 2012 – Last-patient-last-visit 26 July 2013

Countries: Brazil, South Africa, Tanzania

Arms:

- Randomized:
  - DS-TB: $Pa_{100}M_{400}Z_{1500}$ once daily for 8 weeks (n = 60)
  - DS-TB: $Pa_{200}M_{400}Z_{1500}$ once daily for 8 weeks (n = 62)
  - DS-TB: HRZE for 8 weeks (n = 59)
- Non-randomized:
  - MDR-TB: $Pa_{200}M_{400}Z_{1500}$ once daily for 8 weeks (n = 26)

From Reference 1: "In patients with drug-susceptible tuberculosis, the bactericidal activity of $MPa_{200}Z$ (n=54) on days 0–56 (0·155, 95% Bayesian credibility interval 0·133–0·178) was significantly greater than for HRZE (n=54, 0·112, 0·093–0·131). DR-$MPa_{200}Z$ (n=9) had bactericidal activity of 0·117 (0·070–0·174). The bactericidal activity on days 7–14 was strongly associated with bactericidal activity on days 7–56. Frequencies of adverse events were similar to standard treatment in all groups. The most common adverse event was hyperuricaemia in 59 (29%) patients (17 [28%] patients in $MPa_{100}Z$ group, 17 [27%] patients in $MPa_{200}Z$ group, 17 [29%] patients in HRZE group, and 8 [31%] patients in DR-$MPa_{200}Z$ group). Other common adverse events were nausea in (14 [23%] patients in $MPa_{100}Z$ group, 8 [13%] patients in $MPa_{200}Z$ group, 7 [12%] patients in HRZE group, and 8 [31%] patients in DR-$MPa_{200}Z$ group) and vomiting

(7 [12%] patients in $MPa_{100}Z$ group, 7 [11%] patients in $MPa_{200}Z$ group, 7 [12%] patients in HRZE group, and 4 [15%] patients in DR-$MPa_{200}Z$ group). No on-treatment electrocardiogram occurrences of corrected QT interval more than 500 ms (an indicator of potential of ventricular tachyarrhythmia) were reported. No phenotypic resistance developed to any of the drugs in the regimen."

NC-005

Title: A Phase 2 Open-Label Partially Randomized Trial to Evaluate the Efficacy, Safety and Tolerability of Combinations of Bedaquiline, Moxifloxacin, PA-824 and Pyrazinamide During 8 Weeks of Treatment in Adult Subjects with Newly Diagnosed Drug-Sensitive or Multi Drug-Resistant, Smear-Positive Pulmonary Tuberculosis[2]

Dates: First-patient-first-visit 23 October 2014 – Last-patient-last-visit 23 January 2018

Countries: South Africa, Tanzania, Uganda

Arms:

- Randomized:
    - DS-TB: $B_{load}Pa_{200}Z_{1500}$ for 8 weeks[1] (n = 59[2])
    - DS-TB: $B_{200}Pa_{100}Z_{1500}$ once daily for 8 weeks (n = 60)
    - DS-TB: HRZE for 8 weeks (n = 61)
- Not randomized:
    - MDR-TB: $B_{200}Pa_{200}M_{400}Z_{1500}$ once daily for 8 weeks (n = 60)

[1]B_{load} = 400 mg once daily for 2 weeks then 200 mg thrice weekly for 6 weeks. Pa_{200} and Z_{1500} once daily for 8 weeks.

[2]One participant with no post-baseline ALT measurements was excluded from the analysis here, hence the sample size for BPaZ in Table 1 is 118, not 119.

From Reference 2: "57 patients in the $B_{load}PaZ$ group, 56 in the $B_{200}PaZ$ group, and 59 in the HRZE group were included in the primary analysis. $B_{200}PaZ$ produced the highest daily percentage change in TTP (5·17% [95% Bayesian credibility interval 4·61–5·77]), followed by $B_{load}PaZ$ (4·87% [4·31–5·47]) and HRZE group (4·04% [3·67–4·42]). The bactericidal activity in $B_{200}PaZ$ and $B_{load}PaZ$ groups versus that in the HRZE group was significantly different. Higher proportions of patients in the $B_{load}PaZ$ (six [10%] of 59) and $B_{200}PaZ$ (five [8%] of 60) groups discontinued the study drug than in the HRZE group (two [3%] of 61) because of adverse events. Liver enzyme elevations were the most common grade 3 or 4 adverse events and resulted in the withdrawal of ten patients (five [8%] in the $B_{load}PaZ$ group, three [5%] in the $B_{200}PaZ$ group, and two [3%] in the HRZE group). Serious treatment-related adverse events affected two (3%) patients in the $B_{load}PaZ$ group and one (2%) patient in the HRZE group. Seven (4%) patients with drug-susceptible tuberculosis died and four (7%) patients with rifampicin-resistant tuberculosis died. None of the deaths were considered to be related to treatment."

STAND

Title: A Phase 3 Open-Label Partially Randomized Trial to Evaluate the Efficacy, Safety and Tolerability of the Combination of Moxifloxacin plus PA-824 plus Pyrazinamide after 4 and 6 months of Treatment in Adult Subjects with Drug-Sensitive Smear-Positive Pulmonary Tuberculosis and after 6 months of Treatment in Adult Subjects with Multi-Drug Resistant, Smear-Positive Pulmonary Tuberculosis[3]

Dates: First-patient-first-visit 19 February 2015 – Last-patient-last-visit 29 November 2017

Countries: Georgia, Kenya, Malaysia, Philippines, South Africa, Thailand, Uganda, Ukraine, Tanzania

Arms:

- Randomized:
    - DS-TB: $Pa_{100}M_{400}Z_{1500}$ once daily for 4 months (n = 65)
    - DS-TB: $Pa_{200}M_{400}Z_{1500}$ once daily for 4 months (n = 71)
    - DS-TB: $Pa_{200}M_{400}Z_{1500}$ once daily for 6 months (n = 67)
    - DS-TB: HRZE for 2 months/HR for 4 months (n = 68)
- Not randomized:
    - MDR-TB: $Pa_{200}M_{400}Z_{1500}$ once daily for 6 months (n = 13)

From Reference 3: "Respectively 4/47 (8.5%), 11/57 (19.3%), 14/52 (26.9%) and 1/53 (1.9%) DS-TB outcomes were unfavourable in patients on $6Pa_{200}MZ$, $4Pa_{200}MZ$, $4Pa_{100}MZ$ and controls. There was a 6.6% (95% CI –2.2% to 15.4%) difference per protocol and 9.9% (95%CI –4.1% to 23.9%) modified intention-to-treat difference in unfavourable responses between the control and $6Pa_{200}MZ$ arms. Grade 3 adverse events affected 68/203 (33.5%) receiving experimental regimens, and 19/68 (27.9%) on control. Ten of 203 (4.9%) participants on experimental arms and 2/68 (2.9%) controls died."

Nix-TB

Title: A Phase 3, Open-label Trial Assessing the Safety and Efficacy of Bedaquiline Plus Pretomanid Plus Linezolid in Subjects with Pulmonary Infection of Either Extensively Drug-resistant Tuberculosis (XDR-TB) or Treatment Intolerant/Non-responsive Multi-Drug Resistant [Tuberculosis] (MDR-TB)[4]

Dates: First-patient-first-visit 16 April 2015 – Last-patient-last-visit 3 August 2020

Country: South Africa

Arms:

- Not randomized:
    - DR-TB[1]:$B_{load}Pa_{200}L_{1200}$ for 26 weeks[2,3] (n = 109)

[1]See Title for types of DR-TB.

[2]B_{load} = 400 mg once daily for 2 weeks then 200 mg thrice weekly for 24 weeks. Pa_{200} once daily for 26 weeks.

[3]L_{1200} initially as once daily then amended to 600 mg twice daily.

From Reference 4: “At 6 months after the end of treatment in the intention-to-treat analysis, 11 patients (10%) had an unfavorable outcome and 98 patients (90%; 95% confidence interval, 83 to 95) had a favorable outcome. The 11 unfavorable outcomes were 7 deaths (6 during treatment and 1 from an unknown cause during follow-up), 1 withdrawal of consent during treatment, 2 relapses during follow-up, and 1 loss to follow-up. The expected linezolid toxic effects of peripheral neuropathy (occurring in 81% of patients) and myelosuppression (48%), although common, were manageable, often leading to dose reductions or interruptions in treatment with linezolid.”

ZeNix

Title: A Phase 3 Partially-blinded, Randomized Trial Assessing the Safety and Efficacy of Various Doses and Treatment Durations of Linezolid Plus Bedaquiline and Pretomanid in Participants with Pulmonary Infection of Either Extensively Drug-resistant Tuberculosis (XDR-TB), Pre-XDR-TB or Treatment Intolerant or Non-responsive Multi-drug Resistant Tuberculosis (MDR-TB)[5]

Dates: First-patient-first-visit 16 November 2017 – Last-patient-last-visit 26 November 2021

Countries: Georgia, Moldova, Russia, South Africa

Arms:

- Randomized:
    - DR-TB[1]: Linezolid 1200 mg for 26 weeks: $B_{QD}Pa_{200}L_{1200}$ once daily for 26 weeks[2] (n = 45)
    - DR-TB: Linezolid 1200 mg for 9 weeks: $B_{QD}Pa_{200}$ once daily for 26 weeks plus L_{1200} once daily for 9 weeks.
    - DR-TB: Linezolid 600 mg for 26 weeks: $B_{QD}Pa_{200}L_{600}$ once daily for 26 weeks (n = 45)
    - DR-TB: Linezolid 600 mg for 9 weeks: $B_{QD}Pa_{200}$ once daily for 26 weeks plus L_{600} once daily for 9 weeks. (n = 45)

[1]See Title for types of DR-TB.

[2]B_{QD} = 200 mg once daily for 8 weeks then 100 mg once daily for 18 weeks.

From Reference 5: “Among participants who received bedaquiline–pretomanid–linezolid with linezolid at a dose of 1200 mg for 26 weeks or 9 weeks or 600 mg for 26 weeks or 9 weeks, 93%, 89%, 91%, and 84%, respectively, had a favorable outcome; peripheral neuropathy occurred in 38%, 24%, 24%, and 13%, respectively; myelosuppression occurred in 22%, 15%, 2%, and 7%, respectively; and the linezolid dose was modified (i.e., interrupted, reduced, or discontinued) in 51%, 30%, 13%, and 13%, respectively. Optic neuropathy developed in 4 participants (9%) who had received linezolid at a dose of 1200 mg for 26 weeks; all the cases resolved. Six of the seven unfavorable microbiologic outcomes through 78 weeks of follow-up occurred in participants assigned to the 9-week linezolid groups.”

SimpliciTB

Title: An Open-Label, Partially Randomized Trial to Evaluate the Efficacy, Safety and Tolerability of a 4-month Treatment of Bedaquiline plus Pretomanid plus Moxifloxacin plus Pyrazinamide (BPaMZ) Compared to a 6-month Treatment of HRZE/HR (Control) in Adult Participants with Drug-Sensitive Smear-Positive Pulmonary Tuberculosis (DS-TB) and a 6-month Treatment of BPaMZ in Adult Participants with Drug Resistant, Smear-Positive Pulmonary Tuberculosis (DR-TB)[6]

Dates: First-patient-first-visit 23 July 2018 – Last-patient-last-visit 5 April 2022

Countries: Brazil, Georgia, Phillipines, Russia, South Africa, Tanzania, Uganda

Arms:

- Randomized:
  - DS-TB: $B_{QD}Pa_{200}M_{400}Z_{1500}$ once daily for 17 weeks (n = 150)
  - DS-TB: HRZE for 2 months/HR for 4 months (n = 153[1])
- Not randomized:
  - DR-TB[2]: $B_{QD}Pa_{200}M_{400}Z_{1500}$ once daily for 26 weeks (n = 152[3])

[1]One participant with no post-baseline ALT measurements was excluded from the analysis here, hence the sample size for HRZE in Table 1 is 152, not 153.

[2]Participants with DR-TB were defined as mono-resistance to rifampicin or isoniazid, or resistance to both rifampicin and isoniazid (MDR-TB).

[3]Three participants with no post-baseline ALT measurements were excluded from the analysis here, hence the sample size for BPaMZ in Table 1 is 299, not 302.

From Reference 6: “In a modified intention to treat analysis (mITT), by week 8, 122 (84.1%) and 70 (47.3%) participants respectively were culture negative on 4BPaMZ and HRZE with a Hazard Ratio for earlier negative status of 2.93 (95% CI, 2.17-3.96). Median time to negative culture was 6 weeks (IQR: 4-8) on 4BPaMZ and 11 weeks (IQR: 6-12) on HRZE. 152 DR-TB participants received 6BPaMZ with a median time to negative culture of 5 weeks (IQR 3-7). At week 52, 17%, 7% and 17% of participants had unfavourable outcomes on 4BPaMZ, HRZE and 6BPaMZ, respectively. The absolute difference between DS-TB arms was 9.72% (95% CI, 2.35-17.09%) so 4BPaMZ did not meet the non-inferiority margin of 12% in the microbiologically eligible and assessable analysis population (TB-mITT). Higher unfavourable outcome rates on 4BPaMZ were due to withdrawals for adverse events, predominantly related to elevated hepatic enzymes which precluded treatment completion in 7% of participants.”

Additional methodological details

Introduction

The objective of this investigation was to compare three regimen groups – BPaL, PaZX, and HRZE – with respect to the probability of hepatotoxicity over eight weeks of treatment using time-to-event (TTE) analysis.

Data were pooled from six studies conducted by TB Alliance. In chronological order, these studies were: NC-002, NC-005, NC-006 = STAND, Nix-TB, NC-007 = ZeNix, and NC-008 = SimpliciTB.

The duration of treatment was eight weeks in NC-002 and NC-005. In the other studies, the duration of treatment was 17 – 26 weeks.

Hepatotoxicity was identified by elevations of ALT to >3xULN, >5xULN, >8xULN, or >10xULN; and, for each value of xULN, the event of the TTE analysis was either the first occurrence of such an elevation of ALT within eight weeks or censoring.

ALT was measured only when participants visited the clinic. Thus, times to event were essentially bound to the times of clinic visits, which generally occurred at intervals of one or two weeks. All studies had visits scheduled immediately after completing the eighth week of treatment. In NC-002 and NC-005, this Week 8 visit was scheduled to occur on Study Day 57, the morning after the 56th treatment day, and was called the Day 57 visit. In the later, longer studies, the visit was referred to as the Week 8 visit, and a day for it was not specified in the protocol. Here, the term *Week 8 visit* will be used for all studies.

However, not all participants in all studies had a Week 8 visit. Some ended treatment before the Week 8 visit (e.g., withdrew, were withdrawn, were lost to follow-up, died, etc.). Others simply missed the visit, especially in the longer studies, where they may have continued treatment through and beyond Week 8 without providing any ALT data at Week 8.

Participants who did have a Week 8 visit did not always make it to the clinic on Day 57 for that visit, even in NC-002 and NC-005 where that was the nominal intent. Thus, the duration of follow-up through eight weeks varied among participants who had treatment through eight weeks.

As stated above, the time span of interest here was eight weeks *of treatment*. Treatment may have stopped before eight weeks, and may not have stopped on a visit day, whereas events could operationally occur only on visit days. The effect, if any, of a treatment on the liver may be expected to extend beyond the exact day of treatment cessation.

Thus, conventions were needed for how to handle missed Week 8 visits, variation in follow-up time through eight weeks, and what it means to be “on treatment” for the purpose of liver toxicity assessment. Here we describe the conventions used.

Conventions

Missing Week 8 visit

A subject who had an event at a visit before Week 8 was counted as such, was removed from the risk set for all later event times, and thus caused no problems.

But for a subject who had no event through the latest visit prior to Week 8 and then continued treatment but did not have a Week 8 visit, it was uncertain whether they should be counted in the risk set for events at Week 8. Therefore, adjustments to the data were made according to the following decision rule:

- Day 50 is identified as the boundary between "before Week 8" and "on or after Week 8". That is, absent a Week 8 visit, a visit occurring on Day ≤ 50 was considered before Week 8, and a visit occurring on a Day > 50 was considered on or after Week 8.
- If the next visit after Day 50 occurred on or before Day 63, that visit was identified as the terminal visit for the Week 8 analysis, and the subject was considered to have completed through Week 8.
- Otherwise, the latest visit up to and including Day 50 was identified as the terminal visit for the Week 8 analysis, and the subject was considered not to have completed through Week 8 (for the purposes of the Week 8 analysis).
- In either case, if the subject had no event through the terminal visit for the Week 8 analysis, they were censored at that visit for the Week 8 analysis.

Variation in "eight weeks"

All visits recognized formally in the source data as Week 8 visits occurred on or after Day 51.

There were 11 subjects for whom visits recognized formally in the source data as Week 8 visits occurred later than Day 61. These were Days 64, 65, 66, 67, 68, 71, 71, 85, 87, 87, and 93.

Thus, only 11 of 1470 subjects in the data pool had Week 8 visits outside of 51 – 61 days.

The existing variability was simply accepted as is. The duration of interest was referred to as "eight weeks" without elaboration.

On Treatment

A visit day was considered On Treatment if it occurred no later than seven days after the recorded Treatment End Day.

Narratives of deaths in the PaZX group attributed to adverse liver events

Case 1

44-year-old female, HIV positive, DS-TB, STAND, Pa_{200}MZ. Illness Onset: Day 23. Death Day: 28. No other hepatotoxins identified. No autopsy performed.

Initial symptoms were nausea and vomiting, managed with proton pump inhibitor and IV fluid replacement. On Day 25, hyperkalaemia treated with IV calcium gluconate and IV actrapid insulin & 50% dextrose. Patient stopped taking IMP as vomiting. On Day 27, ALT: 7124 U/L, total bilirubin: 168 µmol/L. (These results returned from the lab after death.) No recorded treatment pauses before halting.

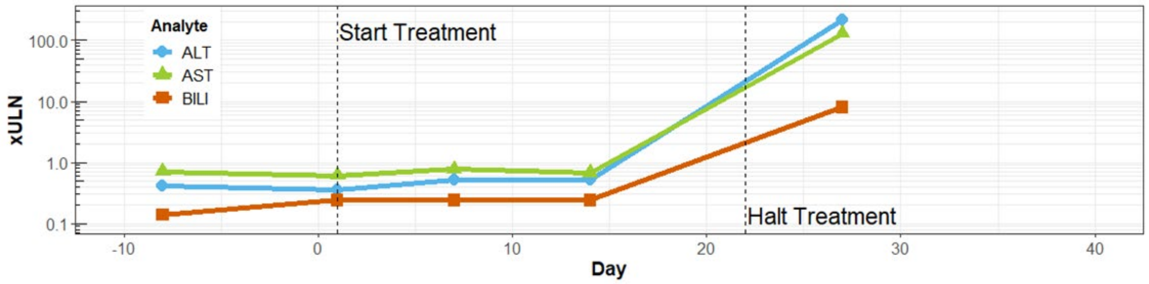

Case 2

21 yrs, male, HIV negative, DS-TB, STAND, Pa_{100}MZ. Illness Onset: Day 21. Death Day: 39. No other hepatotoxins identified. Autopsy performed[1].

Initial symptoms of gastroenteritis, managed supportively without antibiotics. Renal and hepatic biochemistry normal on Day 22 but on Day 29 ALT: 1393 U/L, total bilirubin: 26 µmol/L, and on Day 34 ALT: 3615 U/L, total bilirubin: 185 µmol/L, K: 6.8 mmol/L, creatinine: 206 µmol/L. Patient hospitalised and study medication stopped. Hyperkalaemia treated with IV actrapid insulin & 50% dextrose. IV fluid replacement initiated. Confusion, attributed to hepatic encephalopathy developed after hospitalisation, treated with lactulose. Nutritional support was provided by a dietician. Severe coagulopathy (prothrombin time >120 seconds) was noted on Day 35. Fresh frozen plasma and Vitamin K were administered, alongside a proton pump inhibitor (pantoprazole) and antibiotics (metronidazole). Despite best supportive care, the patient continued to gradually deteriorate with no improvement in the encephalopathy or coagulopathy, both of which were attributed to acute liver failure. No recorded treatment pauses before halting.

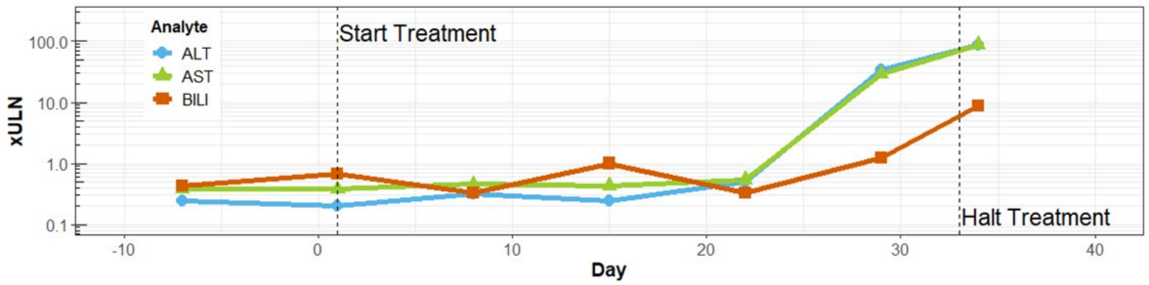

Case 3

23 yrs, female, HIV positive, DS-TB, STAND, Pa$_{200}$MZ. Illness Onset: Day 14. Death Day: 34. No other hepatotoxins identified. No autopsy performed.

Chronic anaemia at baseline (Hb: 6.9 g/dL on screening bloods). Vomiting on Day 14 and 21 but settled each time and felt otherwise well. Hospitalised from Day 25-29 (trial center not informed). On Day 25, ALT: 1649 U/L, total bilirubin: 31 μmol/L, Hb: 6.7 g/dL. Hospital administered transfusion for anaemia. She then developed a rash a few days subsequently that was diagnosed as a transfusion reaction. Re-admitted on Day 32, with confusion, rash, and jaundice. ALT: 2053 U/L, total bilirubin: 171 μmol/L. A formal decision to discontinue all trial medication was taken, and supportive care was given including IV antibiotics (ceftriaxone) and lactulose to manage hepatic encephalopathy. The patient progressively deteriorated and died. Lab values on Days 25 and 32 were obtained from local laboratory and hard-coded into the analysis dataset. No recorded treatment pauses before halting.

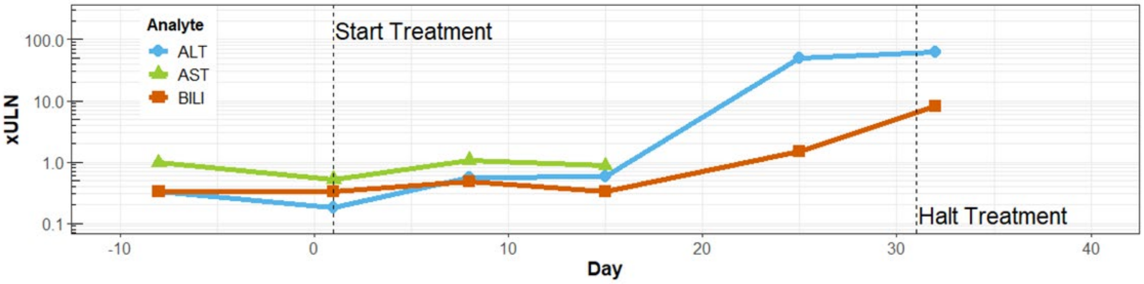

Case 4

54 yrs, male, HIV negative, DS-TB, SimplicTB, BPa$_{200}$MZ. Illness Onset: Day 28. Death Day: 48. Other hepatotoxins: Alcohol, including locally brewed gongo (revealed retrospectively by family)[2]. Autopsy performed[3].

On Day 28, asymptomatic ALT and AST elevation were noted (71 U/L and 174 U/L). Total bilirubin was 7 μmol/L. On Day 31, ALT: 106 U/L, AST: 243 U/L, total bilirubin: 9 μmol/L. Viral hepatitis serology indicated prior hepatitis A (IgG positive) but no evidence of Hepatitis B or C. On Day 33, he had abdominal pain and diarrhoea. ALT: 321 U/L, AST: 1228 U/L, total bilirubin: 7 μmol/L. All study drugs were stopped. On Day 37, ALT: 948 U/L, AST: 2229 U/L, total bilirubin: 112 umol/L. Thrombocytopenia (platelets: 85x109 cells/L). He was hospitalised and treated supportively, including IV fluids and antibiotics (co-amoxiclav). He developed reduced consciousness, coagulopathy, and deranged kidney function. Despite transfer to the Intensive Therapy Unit for mechanical ventilation, inotropic support, change of antibiotics to IV meropenem, transfusion of fresh frozen plasma and administration of vitamin K, his deterioration continued. No recorded treatment pauses before halting.

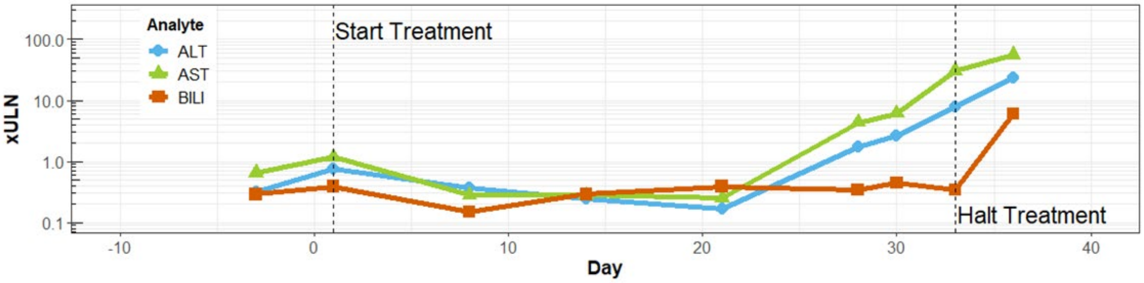

[1]Summary of autopsy result: Tissue sampling from the liver showed extensive autolysis, with no features of granulomatous inflammation, or mass lesions. There was no evidence of interstitial or neoplastic infiltrates, and no vasculitis. Thoracic sections showed left sided pleural effusion, pulmonary oedema, and pneumonia. Kidney samples showed extensive infarction of renal tubules and congestion of renal blood vessels.

[2]Gongo is a highly concentrated, informally brewed alcoholic drink that can contain many impurities including aflatoxin B1 and mycotoxins produced by *Aspergillus flavus* and *Aspergillus parasiticus*. The patient initially denied any consumption of alcoholic drinks but, at a later date, family members and other contacts confirmed that he was repeatedly seen at a local club where Gongo was sold, and his consumption may have been denied because the product was illegal.

[3]Summary of autopsy result: Gross examination of the liver revealed hepatomegaly, micro nodules on the liver, yellowish in color with ascities [ascites]; yellowish fluid. Histologic examination showed fatty liver with distractions of hepatocytes. Gross examination of the kidneys grossly showed "no obvious abnormalities other than mild kidney enlargement." Renal pathology showed necrosis of tubular lining with cells desquamation and dilated proximal tubule, with acute tubular necrosis and marked glomerular necrosis. Toxicology results indicated the presence of metronidazole in liver, kidney, spleen, lung, and blood samples, consistent with treatment the participant received during hospitalization. Blood alcohol test was negative. A second pathologist was asked to review these findings but requested additional information which could not be provided. An independent consultant hepatologist also reviewed this case, noting that: "The most important element… is not chronic disease but acute liver failure, evident by the clinical picture and supported by the findings of both pathologists. Overall, it seems likely that this is acute-on-chronic liver injury resulting in death from liver failure and terminal sepsis. The probable cause is one or more drugs in the pretomanid/bedaquiline/moxifloxacin/pyrazinamide regimen, and a contribution of alcohol injury and even aflatoxin hepatotoxicity cannot be excluded, based on available evidence."

Figures

Figure S1: Time to First Elevation of More Than 3xULN / 5xULN / 8xULN / 10xULN by Regimen Group

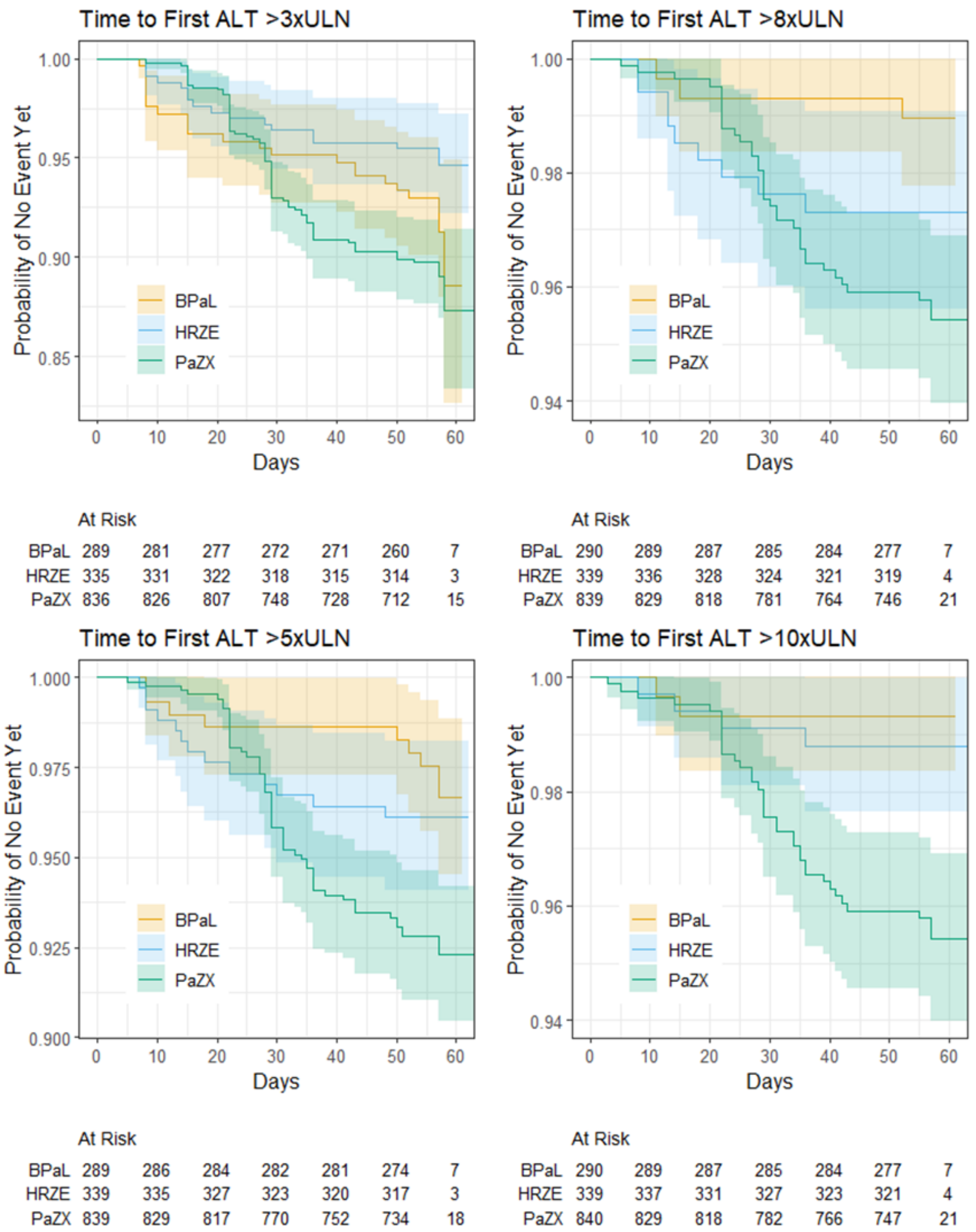

Tables

Table S1: Scheduled Visits for Laboratory Safety Tests Per Protocol Post Randomization

TB Alliance completed studies (treatment ≥8 weeks)		Day 1	Week 1	Week 2	Week 3	Week 4	Week 5	Week 6	Week 7	Week 8
Phase 2	NC-002	X	X	X	X	X	X	X	X	X
	NC-005	X	X	X	X	X	X	X	X	X
	SimpliciTB	X	X	X	X	X	X	X	X	X
Phase 3	NC-006	X	X	X		X				X
	Nix-TB	X	X	X		X		X		X
	ZeNix	X	X	X	X	X		X		X

Note: Investigators had the discretion to request unscheduled visit at any time during the study conduct.

Table S2: Participant Flow Through Outcomes

Table S2a: >3xULN

Outcome, by regimen group

Outcome	HRZE	PaZX	BPaL
Potentially Eligible	340	840	290
Ineligible, Baseline >3xULN	-5	-4	-1
Initial Risk Set	335	836	289
Treatment-Emergent Event	17	88	25
Completed, No Event	310	701	253
Did Not Complete, No Event	8	47	11

Early withdrawal reasons for “Did Not Complete, No Event”, by regimen group

Early.Withdrawal.Reason	HRZE	PaZX	BPaL
Adverse Event	0	11	0
Death	0	3	4
Late Exclusion	1	12	1
Lost to Follow-Up	0	0	1
Missed Week 8 Visit	0	6	4
Non-compliance	1	0	0
Other	2	3	0
Physician or Sponsor Decision	0	6	0
Protocol Deviation	1	2	0
Withdrawal of Consent	3	2	1
<NA>	0	2	0

Table S2b: >5xULN

Outcome, by regimen group

Outcome	HRZE	PaZX	BPaL
Potentially Eligible	340	840	290
Ineligible, Baseline >5xULN	-1	-1	-1
Initial Risk Set	339	839	289
Treatment-Emergent Event	13	61	9
Completed, No Event	316	723	269
Did Not Complete, No Event	10	55	11

Early withdrawal reasons for “Did Not Complete, No Event”, by regimen group

Early.Withdrawal.Reason	HRZE	PaZX	BPaL
Adverse Event	2	18	0
Death	0	3	4
Late Exclusion	1	12	1
Lost to Follow-Up	0	0	1
Missed Week 8 Visit	0	6	4
Non-compliance	1	0	0
Other	2	4	0
Physician or Sponsor Decision	0	6	0
Protocol Deviation	1	2	0
Withdrawal of Consent	3	2	1
<NA>	0	2	0

Table S2c: >8xULN

Outcome, by regimen group

Outcome	HRZE	PaZX	BPaL
Potentially Eligible	340	840	290
Ineligible, Baseline >8xULN	-1	-1	0
Initial Risk Set	339	839	290
Treatment-Emergent Event	9	36	3
Completed, No Event	318	739	276
Did Not Complete, No Event	12	64	11

Early withdrawal reasons for “Did Not Complete, No Event”, by regimen group

Early.Withdrawal.Reason	HRZE	PaZX	BPaL
Adverse Event	4	26	0
Death	0	3	4
Late Exclusion	1	12	1
Lost to Follow-Up	0	0	1
Missed Week 8 Visit	0	6	4
Non-compliance	1	0	0
Other	2	4	0
Physician or Sponsor Decision	0	6	0
Protocol Deviation	1	2	0
Regimen Interruption	0	1	0
Withdrawal of Consent	3	2	1
<NA>	0	2	0

Table S2d: >10xULN

Outcome, by regimen group

Outcome	HRZE	PaZX	BPaL
Potentially Eligible	340	840	290
Ineligible, Baseline >10xULN	-1	0	0
Initial Risk Set	339	840	290
Treatment-Emergent Event	4	36	2
Completed, No Event	320	740	277
Did Not Complete, No Event	15	64	11

Early withdrawal reasons for “Did Not Complete, No Event”, by regimen group

Early.Withdrawal.Reason	HRZE	PaZX	BPaL
Adverse Event	7	26	0
Death	0	3	4
Late Exclusion	1	12	1
Lost to Follow-Up	0	0	1
Missed Week 8 Visit	0	6	4
Non-compliance	1	0	0
Other	2	4	0
Physician or Sponsor Decision	0	6	0
Protocol Deviation	1	2	0
Regimen Interruption	0	1	0
Withdrawal of Consent	3	2	1
<NA>	0	2	0

Table S3: Cox Model for Time to First Elevation More than 8xULN, HRZE versus PaZX versus BPaL

Variable	Coef	Haz. Ratio	se(Coef)	z	p-value
HRZE(Initial):PaZX	2.03	7.58	1.01	2.00	0.046
HRZE(Final):PaZX	-2.19	0.112	0.942	-2.33	0.02
BPaL:PaZX	-1.86	0.156	0.615	-3.02	0.003
HRZE(Initial):BPaL	3.88	48.6	1.13	3.44	0.001
HRZE(Final):BPaL	-0.335	0.716	1.12	-0.300	0.765
Baseline(Initial)	2.20	9.00	0.430	5.11	< 0.001
Baseline(Final)	-0.103	0.902	0.429	-0.239	0.811
Age	0.0377	1.04	0.0117	3.21	0.001
Weight	0.0109	1.01	0.0111	0.984	0.325
Female	1.15	3.15	0.311	3.69	< 0.001
NonBlack	0.759	2.14	0.337	2.25	0.024
HIVPos	0.298	1.35	0.381	0.781	0.435

Table S4: Cox Model for Time to First Elevation More than 3xULN, PaZX-DS versus PaZX DR versus BPaL

Variable	Coef	Haz. Ratio	se(Coef)	Z	p-value
PaZX-DS:PaZX-DR	0.599	1.82	0.270	2.22	0.026
BPaL:PaZX-DR	-0.176	0.839	0.320	-0.549	0.583
PaZX-DS:BPaL	0.775	2.17	0.255	3.03	0.002
Baseline	0.648	1.91	0.186	3.48	0.001
Age	0.0166	1.02	0.00820	2.02	0.043
Weight	0.0142	1.01	0.00731	1.94	0.052
Female	0.427	1.53	0.209	2.04	0.042
NonBlack	0.506	1.66	0.223	2.27	0.023
HIVPos	0.308	1.36	0.253	1.22	0.224
AUC	0.00137	1.00	0.00321	0.427	0.670

Table S5: Cox Model for Time to First Elevation More than 8xULN, PaZX-DS versus PaZX-DR versus BPaL

Variable	Coef	Haz. Ratio	se(Coef)	z	p-value
PaZX-DS:PaZX-DR	1.45	4.28	0.537	2.71	0.007
BPaL:PaZX-DR	-1.04	0.352	0.871	-1.20	0.231
PaZX-DS:BPaL	2.50	12.1	0.740	3.38	0.001
Baseline	0.254	1.29	0.382	0.665	0.506
Age	0.0344	1.04	0.0132	2.61	0.009
Weight	0.0192	1.02	0.0124	1.54	0.123
Female	1.13	3.11	0.352	3.23	0.001
NonBlack(Initial)	3.92	50.6	1.54	2.54	0.011
NonBlack(Final)	-0.194	0.823	0.641	-0.303	0.762
HIVPos	0.00704	1.01	0.486	0.0145	0.988
AUC	0.00644	1.01	0.00496	1.30	0.194

Table S6: Cox Model for Time to First Elevation More than 3xULN, HRZE versus PaZX-DS

Variable	Coef	Haz. Ratio	se(Coef)	z	p-value
HRZE:PaZX-DS	-0.855	0.425	0.271	-3.15	0.002
Baseline	0.398	1.49	0.242	1.65	0.099
Age	0.0187	1.02	0.00904	2.07	0.039
Weight	0.0171	1.02	0.00794	2.15	0.031
Female	0.435	1.55	0.232	1.88	0.06
NonBlack(Initial)	2.14	8.50	0.719	2.98	0.003
NonBlack(Final)	-0.0754	0.927	0.432	-0.175	0.861
HIVPos	0.740	2.10	0.265	2.80	0.005

Table S7: Cox Model for Time to First Elevation More than 8xULN, HRZE versus PaZX-DS

Variable	Coef	Haz. Ratio	se(Coef)	z	p-value
HRZE(Initial):PaZX-DS	4.35	77.5	1.69	2.57	0.010
HRZE(Final):PaZX-DS	-1.70	0.183	0.471	-3.61	< 0.001
Baseline(Initial)	2.33	10.3	0.447	5.20	< 0.001
Baseline(Final)	-0.147	0.863	10.7	-0.0137	0.989
Age	0.0306	1.03	0.0106	2.89	0.004
Weight	0.00920	1.01	0.0102	0.898	0.369
Female	0.788	2.20	0.273	2.89	0.004
NonBlack	0.772	2.16	0.301	2.57	0.010
HIVPos	0.903	2.47	0.314	2.88	0.004

Table S8: Treatment-Emergent Potential Hy's Law Cases

Study	HRZE	PaZX	BPaL
NC-002	1 (1.7%)		
NC-005	1 (1.6%)	1 (0.56%)	
STAND	1 (1.5%)	5[a,b] (2.3%)	
Nix-TB	-	-	1[c] (0.92%)
ZeNix	-	-	1[d] (0.55%)
SimpliciTB	0 (0%)	1 (0.33%)	
Total	3 (0.88%)	7 (0.83%)	2 (0.69%)

[a]Includes one subject who was discontinued from $Pa_{200}MZ$ on Day 125 and started on HRZE with the NTP on Day 154; the first occurrence of BILI > 2xULN was Day 186.
[b]Includes one subject whose ALT and BILI elevations were reported only by local labs, not contained in study central lab database, but were hard-coded into this analysis dataset. See Narrative Case 3.
[c]This participant had a medical history of alcohol use. Potential-Hy's-Law lab criteria were met per central lab results at Week 8, with ALP 1.8 xULN. A local lab repeated 4 days later showed ALP 2.5 xULN. The BPaL regimen was rechallenged later, and the participant was able to complete the remaining 4 months of treatment (6-month full treatment course) with no issues.
[d]This participant met the potential-Hy's-Law lab criteria at study Day 183, which was attributed to documented acute hepatitis B.

Hepatotoxicity Guidelines

NC-002

Protocol Section 7.3.1. ALT and AST

Management will be at the discretion of the Investigator, according to generally accepted medical practice standards.

Grade 1 (> 1.0 to < 2.0 x ULN), Grade 2 (⩾ 2.0 to < 3.0 x ULN) AST or ALT elevation:

Participants may continue IMP. Participants should be followed until resolution (return to baseline) or stabilization of AST/ALT elevation.

Grade 3 (⩾ 3.0 to ⩽ 8.0 x ULN) or Grade 4 (> 8.0 x ULN) AST or ALT elevation:

Participants will permanently discontinue IMP and be withdrawn from the trial. It is recommended that the investigator contacts the sponsor to discuss the case of AST or ALT elevation. Participants should be followed until resolution (return to baseline) or stabilization of AST/ALT elevation.

NC-005

Protocol Section 7.3.1. ALT and AST

> 1.0 to < 5.0 x ULN AST or ALT elevation:

Management will be at the discretion of the Investigator, according to generally accepted medical practice standards, and Subjects may continue IMP. Subjects should be followed until resolution (return to baseline) or stabilization of AST/ALT elevation.

> 5.0 x ULN or Grade 4 (> 8.0 x ULN) AST or ALT elevation:

Subjects will permanently discontinue IMP and be withdrawn from the trial. It is recommended that the Investigator contacts the Sponsor to discuss the case of AST or ALT elevation. Subjects should be followed until resolution (return to baseline) or stabilization of AST/ALT elevation.

STAND

Protocol Section 7.3.1. Hepatic Toxicity

Management of hepatic toxicity is described in Appendix 9.

Protocol Appendix 9 LIVER TOXICITY MANAGEMENT

Standard anti TB chemotherapy is known to cause derangement of liver function tests in a substantial number of patients. In many cases this will be asymptomatic and self-limiting. In some cases severe hepatitis and even fulminant liver failure and death can occur.

In pre-marketing clinical trials of new drugs and regimens it is especially important to identify and carefully manage any trial subjects who are at risk of progression to serious liver injury. The observation of altered liver function to a degree that has a high risk of progressing to liver failure has been referred to informally as *Hy's Law* (Temple 2001; Reuben 2004); this reflects that pure hepatocellular injury sufficient to cause hyperbilirubinemia is an ominous indicator of the potential for a drug to cause serious liver injury. Briefly, Hy's Law cases have the following three components:

1. The drug causes hepatocellular injury, generally shown by a higher incidence of 3 fold or greater elevations above the ULN of ALT or AST than the (non hepatotoxic) control drug or placebo

2. Among trial subjects showing such aminotransferase (AT) elevations, often with ATs much greater than 3xULN, one or more also show elevation of serum total bilirubin (TBL) to >2xULN, without initial findings of cholestasis (elevated serum ALP)

3. No other reason can be found to explain the combination of increased AT and TBL, such as viral hepatitis A, B, or C; pre existing or acute liver disease; or another drug capable of causing the observed injury

During the trial, liver function will be monitored regularly with clinical assessment and blood tests in study participants and this will assist in follow up laboratory measurements that can document either resolution of abnormalities or signal the potential for drug induced liver injury (DILI). In a clinical trial of new drugs and combinations it is especially important for investigators to follow closely any subjects who have evidence of hepatic inflammation or potential toxicity. The following procedure describes the management of deranged liver function tests in study participants.

Procedure

Blood tests for liver function will be taken routinely at screening (Day 9 to 1) and at the specific time points designated in the protocol, and at Early Withdrawal. If at any other visit the clinician suspects derangement of liver function, e.g. the subject describes nausea and vomiting, right upper abdominal pain or is jaundiced, blood should be taken for liver function tests and the subject comprehensively assessed for evidence of hepatitis or hepatic impairment and any potentially contributing causes.

The laboratory source (print out of any results) should be stored alongside or transcribed into the clinical source document. Each abnormal value should be marked as clinically significant (CS) or non clinically significant (NCS); the assessment of significance is at the discretion of the investigator. All clinically significant abnormal results must be recorded as Adverse Events in the e CRF and graded clinically as per the DMID adult toxicity table grading. Assessments and decision making for elevations in aminotransferase values or bilirubin of various levels of concern are detailed below:

Decision to Consider Stopping Drug Regimen Administration

Consideration of stopping drug administration, at least temporarily, to subjects with liver function abnormalities or signs and symptoms of hepatitis should be discussed with the sponsor medical monitor, especially in the following situations:

- ALT or AST >8xULN
- ALT or AST >5xULN for more than 2 weeks
- ALT or AST >3xULN with the appearance of fatigue, nausea, vomiting, right upper quadrant pain or tenderness, fever, rash, and/or eosinophilia (>5%)

The drug regimen should be interrupted and the subject's clinical course should be discussed with the sponsor medical monitor in the following situation:

- ALT or AST >3xULN and Total Bilirubin >2xULN

More detailed assessments and decision making for elevations in aminotransferase values or bilirubin of various levels of concern are detailed below:

Grade 3 (DMID grading: ALT, AST, AP greater than or equal to 3X ULN to 8ULN if a substantial increase from baseline (such as > 2 fold increase):

Contact the subject and recall them as soon as possible. Assess the subject for other signs and symptoms of more specific hepatic events including hepatic impairment and or hepatitis. If you are concerned you should consider arranging for the subject to present to a medical facility (e.g. emergency department) immediately for assessment.

- Assess the clinical significance the subject has jaundice, a coagulation disorder or signs of hepatic encephalopathy. All study drugs should be withheld pending assessment/improvement.

- Assess possible contributing factors This should include (but is not limited to) alcohol, intra venous and other drug use, travel, unwell contacts, any medications with known hepatotoxic potential, herbal products and dietary supplements, previous or known hepatitis infection and exposure to environmental chemical agents. Although anti TB chemotherapy is known to cause liver function test derangement, the subject should always be assessed for other possible causes or contributing factors.
- The subject should also be advised to stop taking any medications/substances, other than the study medications to treat TB, that may be contributing to or causing derangement of liver function tests.
- Make every effort to repeat the testing of ALT, AST, AP and bilirubin within 48 72 hours to confirm the abnormalities and to determine if they are increasing or decreasing. Consider any additional laboratory tests that may help characterize the subjects clinical condition. Subjects should have tests for causes of viral hepatitis (e.g. hepatitis A and B and any other tests available of viral hepatitis). If tests for viral hepatitis are not available or not done it may still be helpful to collect an additional 10ml sample for serum for freezing (5ml yellow/SST tube x2) which may be tested later. The subjects consent must be obtained for this.

Elevated liver enzymes considered of clinical significance, but not accompanied by other signs and symptoms, should be reported as an adverse event and should usually be recorded as elevated liver enzymes. If the term "hepatitis" is used, the Safety Data Manager will question the site for additional evidence to support the diagnosis, such as clinical signs and serological or biopsy data. While a liver biopsy is not required to make a diagnosis of hepatitis, the term "hepatitis" should be reserved in most instances for cases where there is supportive evidence beyond a liver enzyme abnormality. However, if the investigator will confirm the diagnosis of hepatitis just on the basis of clinical signs and laboratory values the diagnosis will be accepted. Should other symptoms or signs be present, these should also be recorded as adverse events.

<u>If liver function tests are Grade 4 (DMID grading: ALT, AST, AP > 8 ULN:</u>

- Contact the subject and recall them as soon as possible. Generally the clinical trial medication should be withdrawn, but this should be discussed first with the sponsor Medical Monitor whenever possible. Assess the subject for other signs and symptoms of more specific hepatic events including hepatic impairment and or hepatitis. If you are concerned you should consider arranging for the subject to present to a medical facility (e.g. emergency department) immediately for assessment.
- Assess the clinical significance Consider hospitalisation if the ALT is more than 10 times the ULN and/or the subject has jaundice, a coagulation disorder or signs of hepatic encephalopathy. All study drugs should be withheld pending assessment/improvement.
- Assess possible contributing factors This should include (but is not limited to) alcohol, intra venous and other drug use, travel, unwell contacts, any medications with known hepatotoxic potential, herbal products and dietary supplements, previous or known hepatitis infection and exposure to environmental chemical agents. Although anti TB chemotherapy is known to cause liver function test derangement, the subject should always be assessed for other possible causes or contributing factors.
- Make every effort to repeat the testing of ALT, AST, AP and bilirubin within 48 72 hours to confirm the abnormalities and to determine if they are increasing or decreasing. Consider any additional laboratory tests that may help characterize the subjects clinical condition. Subjects should have tests for causes of viral hepatitis (e.g. hepatitis A and B and any other tests available of viral hepatitis). If tests for viral hepatitis are not available or not done it may still be helpful to collect an additional 10ml sample for serum for freezing (5ml yellow/SST tube x2) which may be tested later. The subjects consent must be obtained for this.

Elevated liver enzymes considered of clinical significance, but not accompanied by other signs and symptoms, should be reported as an adverse event and should usually be recorded as elevated liver enzymes. If the term "hepatitis" is used, the Safety Data Manager will question the site for additional evidence to support the diagnosis, such as clinical signs and serological or biopsy data. While a liver biopsy is not required to make a diagnosis of hepatitis, the term "hepatitis" should be reserved in most instances for cases where there is supportive evidence beyond a liver enzyme abnormality. However, if the investigator will confirm the diagnosis of hepatitis just on the basis of clinical signs and laboratory values the diagnosis will be accepted. Should other symptoms or signs be present, these should also be recorded as adverse events.

General principles for following subjects with potential liver toxicity

The subject should be contacted regularly depending on the Grade of Liver Function Test elevations and the magnitude of increase relative to baseline values for the subject. Initially this should be daily and subsequently depends on clinical course/individual circumstances. Staff must ensure that all subjects know to seek medical attention urgently if they experience any evidence of worsening liver disease. Symptoms may include (but are not limited to) malaise, fever, nausea, vomiting, loss of appetite, dark urine, yellowing of the eyes or skin (jaundice).

Liver function tests should be repeated regularly, such as every 3 days for the first week then once a week until they return to near baseline values for the subject. Manage the subject symptomatically as required using medications that are not potentially hepatotoxic. Infection control issues must be carefully managed whilst TB medications are being withheld, especially if the subject is still culture positive for acid fast bacilli.

Restarting medication

If the investigator, after consultation with the sponsor medical monitor, stops administration of the study medication, consideration may be given to re starting the study medication. Once the liver function values have decreased substantially and any symptoms have significantly improved a decision must be made about further TB management. This will be dependent on the clinical context and a decision must be made in discussion with the sponsor medical monitor. In all cases treatment should be recommenced under close supervision for any evidence of recurrent liver function abnormalities.

If there is a further significant elevation of hepatic enzymes or bilirubin or symptoms of clinical concern after resumption of study medication, the study medication should be withdrawn permanently. Subjects who permanently discontinue study medication should be managed as clinically indicated according to local National TB Programme guidelines. The sponsor medical monitor can provide advice and examples of suitable treatment regimens to use if required.

Nix-TB

Protocol Section 7.3.1. ALT, AST and Alkaline Phosphatase elevations:

The Investigator should refer to Appendix 8 – Liver Toxicity Management to appropriately manage the Subject for clinically significant elevations of AST, ALT or Alkaline Phosphatase.

Protocol Appendix 8 LIVER TOXICITY MANAGEMENT GUIDELINES

Standard anti-TB chemotherapy is known to cause derangement of liver function tests in a substantial number of patients. In many cases, these will be asymptomatic and self-limiting. In some cases, severe hepatitis and even fulminant liver failure and death can occur.

In pre-marketing clinical trials of new drugs and regimens, it is especially important to identify and carefully manage any trial subjects who are at risk of progressing to serious liver injury. The observation of altered liver function to a degree with a high risk of progressing further to liver failure has been referred to informally as Hy’s Law (Temple 2001; Reuben 2004); this reflects pure hepatocellular injury sufficient to cause hyperbilirubinemia is an ominous indicator of the potential for a drug to cause serious liver injury. Briefly, Hy’s Law cases have the following three components:

1. The drug causes hepatocellular injury, generally shown by a higher incidence of 3-fold or greater elevations above the ULN of ALT or AST than the (non-hepatotoxic) control drug or placebo;

2. Among trial subjects showing such aminotransferase (AT) elevations, often with ATs much greater than 3x ULN, one or more also show elevation of serum total bilirubin (TBL) to >2x ULN, without initial findings of cholestasis (elevated serum ALP) ;

3. No other reason can be found to explain the combination of increased AT and TBL, such as viral hepatitis A, B, or C; pre-existing or acute liver disease; or another drug capable of causing the observed injury.

In a clinical trial of new drugs and combinations, it is especially important for Investigators to closely follow any Subjects who have evidence of potential hepatic inflammation or toxicity. During this trial, liver function will be monitored regularly via clinical assessments and blood tests to assist in determining which follow up laboratory measurements will either document resolution of abnormalities or signal the potential for drug-induced liver injury (DILI). The following procedure describes the management of deranged liver function tests.

<u>Procedure</u>

Blood tests for liver function will be taken routinely at Screening (Days -9 to -1), at the specific visits designated in the protocol and at Early Withdrawal. If at any other visit, the Investigator suspects derangement of liver function (e.g. the Subject describes nausea and vomiting, right upper abdominal pain or is jaundiced), blood should be taken for liver function tests and the Subject comprehensively assessed for evidence of hepatitis, hepatic impairment and any potentially contributing cause(s).

The laboratory source (print-out of any results) should be stored alongside or transcribed into the clinical source document. Each abnormal value should be marked as clinically significant (CS) or non-clinically significant (NCS); the assessment of significance is at the discretion of the Investigator. All abnormal results that are clinically significant must be recorded as Adverse Events in the eCRF and graded clinically per the DMID Adult Toxicity Table (Appendix 2).

Assessments and decision making for elevations in aminotransferase values or bilirubin of various levels of concern are detailed below:

<u>Decision to Consider Stopping Drug Regimen Administration</u>

Consideration of stopping drug administration, at least temporarily, to subjects with liver function abnormalities or signs and symptoms of hepatitis should be discussed with the Sponsor Medical Monitor in the following situations:

- ALT or AST >8x ULN;
- ALT or AST >5x ULN for more than 2 weeks;
- ALT or AST >3x ULN with the appearance of fatigue, nausea, vomiting, right upper quadrant pain or tenderness, fever, rash, and/or eosinophilia (>5%).

> *If a subject has ALT or AST ≥3x ULN **<u>and</u>** Total Bilirubin >2x ULN, the IMP should be interrupted and the Subject's clinical course discussed with the Sponsor Medical Monitor.*

More detailed assessments and decision making for various levels of elevations in aminotransferase values, alkaline phosphatase or bilirubin are detailed below:

Grade 3 per DMID, ALT, AST, AP ULN to 8x ULN or if a substantial increase from baseline (such as > 2-fold increase):

- Contact the Subject and recall them as soon as possible. Assess the Subject for other signs and symptoms of more specific hepatic events including hepatic impairment and/or hepatitis. If you are concerned, you should consider arranging for the subject to present to a medical facility (e.g. emergency department) immediately for assessment.
- Assess the clinical significance - if the Subject has jaundice, a coagulation disorder or signs of hepatic encephalopathy, all study medication should be withheld pending assessment/improvement.
- Assess possible contributing factors – This should include (but is not limited to), alcohol, intra-venous and other drug use, travel, unwell contacts, any medications with known hepatotoxic potential, herbal products and dietary supplements, previous or known hepatitis infection and exposure to environmental chemical agents. Although anti-TB chemotherapy is known to cause liver function test derangement, the Subject should always be assessed for other possible cause(s) or contributing factor(s).
- The Subject should also be advised to stop taking any medications/substances, other than the study medications used to treat TB that may be contributing to or causing derangement of liver function tests.
- Make every effort to repeat the testing of ALT, AST, AP and bilirubin within 48 hours to confirm the abnormalities and determine if they are increasing or decreasing. Consider any additional laboratory tests that may help characterize the Subject's clinical condition. Subjects should be tested for viral hepatitis (e.g. hepatitis A and B and any other tests available of viral hepatitis). If tests for viral hepatitis are not available or done, it may still be helpful to collect an additional 10ml sample for serum for freezing (5ml yellow/SST tube x2) which may be tested later. The Subject's consent must be obtained for this.

Elevated liver enzymes considered to be of clinical significance but not accompanied by other signs and symptoms, should be reported as an adverse event and recorded as elevated liver enzymes in the eCRF. If the term "hepatitis" is used, the Safety Data Manager will question the site for additional evidence to support the diagnosis, such as clinical signs, serological or biopsy data. While a liver biopsy is not required to make a diagnosis of hepatitis, the term "hepatitis" should be reserved in most instances for cases where there is supportive evidence beyond a liver enzyme abnormality. However, if the investigator confirms the diagnosis of hepatitis solely on the basis of clinical signs and laboratory values, the diagnosis will be accepted. Should other symptoms or signs be present, these should also be recorded as adverse events in the eCRF.

If ALT, AST, AP are Grade 4 per DMID (> 8x ULN):

- Contact the Subject and recall them as soon as possible. Generally, the trial medication should be stopped, but this should be discussed first with the Sponsor Medical Monitor whenever possible. Assess the subject for other signs and symptoms of more specific hepatic events, including hepatic impairment and/or hepatitis. If you are concerned, you should consider arranging for the subject to present to a medical facility (e.g. emergency department) immediately for assessment.
- Assess the clinical significance – Consider hospitalisation if the ALT is more than 10 times the ULN and/or the Subject has jaundice, a coagulation disorder or signs of hepatic encephalopathy. All study medications should be withheld pending assessment/improvement.
- Assess possible contributing factors – This should include (but is not limited to), alcohol, intra-venous and other drug use, travel, unwell contacts, any medications with known hepatotoxic potential, herbal products and dietary supplements, previous or known hepatitis infection and exposure to environmental chemical agents. Although anti-TB chemotherapy is known to cause liver function test

derangement, the subject should always be assessed for other possible cause(s) or contributing factor(s).

- Make every effort to repeat the testing of ALT, AST, AP and bilirubin within 48 hours to confirm the abnormalities and determine if they are increasing or decreasing. Consider any additional laboratory tests that may help characterize the subject's clinical condition. Subjects should be tested for viral hepatitis (e.g. hepatitis A and B and any other tests available of viral hepatitis). If tests for viral hepatitis are not available or done, it may still be helpful to collect an additional 10ml sample for serum for freezing (5ml yellow/SST tube x2) which may be tested later. The Subject's consent must be obtained for this.

Elevated liver enzymes considered to be of clinical significance but not accompanied by other signs and symptoms, should be reported as an adverse event and recorded as elevated liver enzymes in the eCRF. If the term "hepatitis" is used, the Safety Data Manager will question the site for additional evidence to support the diagnosis, such as clinical signs, serological or biopsy data. While a liver biopsy is not required to make a diagnosis of hepatitis, the term "hepatitis" should be reserved in most instances for cases where there is supportive evidence beyond a liver enzyme abnormality. However, if the investigator confirms the diagnosis of hepatitis solely on the basis of clinical signs and laboratory values, the diagnosis will be accepted. Should other symptoms or signs be present, these should also be recorded as adverse events in the eCRF.

<u>General Principles for following Subjects with potential liver toxicity</u>

The Subject should be contacted regularly depending on the Grade of LFT elevations and the magnitude of increase relative to baseline for the Subject. Initially, this should be daily and subsequently depends on clinical course/individual circumstances. Staff must ensure all Subjects know to seek medical attention urgently if they experience any evidence of worsening liver disease. Symptoms may include (but are not limited to), malaise, fever, nausea, vomiting, loss of appetite, dark urine, yellowing of the eyes or skin (jaundice).

Liver function tests should be repeated regularly, such as every 3 days for the first week, then once a week until they return to near baseline values for the Subject. Manage the Subject symptomatically as required using medications that are not potentially hepatotoxic. Infection control issues must be carefully managed whilst TB medications are being withheld, especially if the Subject is still culture positive for acid fast bacilli.

<u>Restarting Medication</u>

If the Investigator (after consultation with the Sponsor Medical Monitor), stops administration of the study medication, consideration may be given to re-starting the study medication. Once the liver function values have decreased substantially and symptoms have significantly improved, a decision must be made about further TB management. This will be dependent on clinical context and the decision must be made in discussion with the Sponsor Medical Monitor. In all cases, treatment should be recommenced under close supervision for any evidence of recurrent liver function abnormalities.

If there is a further significant elevation of hepatic enzymes or bilirubin or symptoms of clinical concern after resumption of study medication, the study medication should be withdrawn permanently. Subjects who permanently discontinue study medication should be managed as clinically indicated according to local National TB Programme guidelines. The Sponsor Medical Monitor can provide advice and examples of suitable treatment regimens to use if required.

ZeNix

Protocol Section 8.3.2. ALT, AST and Alkaline Phosphatase elevations:

The Investigator should refer to Appendix 8 – Liver Toxicity Management to appropriately manage the participant for clinically significant elevations of AST, ALT or Alkaline Phosphatase.

Protocol Appendix 8: Liver Toxicity Management

Standard anti-TB chemotherapy is known to cause derangement of liver function tests in a substantial number of patients. In many cases this will be asymptomatic and self-limiting. In some cases, severe hepatitis and even fulminant liver failure and death can occur.

In pre-marketing clinical trials of new drugs and regimens it is especially important to identify and carefully manage any trial participants who are at risk of progression to serious liver injury. The observation of altered liver function to a degree that has a high risk of progressing to liver failure has been referred to informally as Hy's Law;(31,39); this reflects that pure hepatocellular injury sufficient to cause hyperbilirubinemia is an ominous indicator of the potential for a drug to cause serious liver injury. Briefly, Hy's Law cases have the following three components:

1. The drug causes hepatocellular injury, generally shown by a higher incidence of 3-fold or greater elevations above the ULN of ALT or AST than the (non-hepatotoxic) control drug or placebo.

2. Among trial participants showing such aminotransferase (AT) elevations, often with ATs much greater than 3x ULN, one or more also show elevation of serum total bilirubin (TBL) to >2x ULN, without initial findings of cholestasis (elevated serum alkaline phosphatase (ALP)).

3. No other reason can be found to explain the combination of increased AT and total bilirubin level (TBL), such as viral hepatitis A, B, or C; pre-existing or acute liver disease; or another drug capable of causing the observed injury.

During the trial, liver function will be monitored regularly with clinical assessment and blood tests in study participants and this will assist in follow up laboratory measurements that can document either resolution of abnormalities or signal the potential for drug-induced liver injury (DILI). In a clinical trial of new drugs and combinations it is especially important for investigators to follow closely any participants who have evidence of hepatic inflammation or potential toxicity. The following procedure describes the management of deranged liver function tests in study participants.

Procedure

Blood tests for liver function will be taken routinely at screening (Day -9 to -1) and at the specific time points designated in the protocol, and at Early Withdrawal. If at any other visit the clinician suspects derangement of liver function, e.g. the participant describes nausea and vomiting, right upper abdominal pain or is jaundiced, blood should be taken for liver function tests and the participant comprehensively assessed for evidence of hepatitis or hepatic impairment and any potentially contributing causes.

Suspected liver toxicity (or elevated liver enzymes detected in the absence of symptoms) must be taken seriously and detailed guidance will be provided in a separate document "NC-007 Study Management of Hepatotoxicity Guideline". Investigators should refer to this document as a guide to management in cases of suspected or proven liver toxicity. Importantly, the trial Medical Monitor is available to provide further assistance if there is any uncertainty or additional questions.

The laboratory source (print-out of any results) should be stored alongside or transcribed into the clinical source document. Each abnormal value should be marked as clinically significant (CS) or non-clinically significant (NCS); the assessment of significance is at the discretion of the investigator. All clinically significant abnormal results must be recorded as Adverse Events in the eCRF and graded clinically as per the DMID adult toxicity table grading, (Appendix 2). Assessments and decision making for elevations in aminotransferase values or bilirubin of various levels of concern are detailed below:

Elevated liver enzymes considered of clinical significance, but not accompanied by other signs and symptoms, should be reported as an adverse event and should usually be recorded as elevated liver enzymes. If the term "hepatitis" is used, the Safety Data Manager will question the site for additional evidence to support the diagnosis, such as clinical signs and serological or biopsy data. While a liver biopsy is not required to make a

diagnosis of hepatitis, the term "hepatitis" should be reserved in most instances for cases where there is supportive evidence beyond a liver enzyme abnormality. However, if the investigator will confirm the diagnosis of hepatitis just on the basis of clinical signs and laboratory values the diagnosis will be accepted. Should other symptoms or signs be present, these should also be recorded as adverse events.

Restarting Medication

Liver function tests that are improving should be repeated regularly, such as every 3 days for the first week then once a week until they return to near baseline values for the participant. Manage the participant symptomatically as required using medications that are not potentially hepatotoxic. Infection control issues must be carefully managed whilst TB medications are being withheld, especially if the participant is still culture positive for acid fast bacilli.

If medication has been temporarily stopped, once the liver function values have decreased substantially a decision must be made about further TB management. This will be dependent on the clinical context and a decision must be made in discussion with the sponsor medical monitor. Treatment can only be restarted if the trial Medical Monitor is in agreement with the plan. In all cases treatment should be recommenced under close supervision for any evidence of recurrent liver function abnormalities.

If there is a further significant elevation of hepatic enzymes or bilirubin or symptoms of clinical concern after resumption of study medication, the study medication should be withdrawn permanently. Participants who permanently discontinue study medication should be managed as clinically indicated according to local National TB Programme guidelines. The sponsor medical monitor can be contacted for further advice when referring to the National Treatment Program.

The trial Medical Monitor is available to assist the Investigators in both the management of liver toxicity and decisions regarding the holding or re-introduction of trial medication. Investigators must involve the Medical Monitor in any decisions regarding medication hold or re-start, and there should always be a low threshold for contacting the Medical Monitor in cases of elevated liver enzymes.

Hepatotoxicity Management Guideline

1. Introduction

This document is intended to act as a guide for investigators during the management of participants in the ZeNix (NC007) trial with either suspected or biochemically confirmed liver function test abnormalities.

In this document, we intend to provide guidance to the investigators on the selection of patients for enrolment and the management of liver enzyme elevations detected during the course of the trial. **The investigator is responsible for the management of the participant, and this document should not overrule an investigator's clinical judgement but rather support them in their investigation and treatment of the participant.** The study Medical Monitor should be informed as soon as possible if liver toxicity is suspected or confirmed in a participant.

Previous studies have demonstrated the need for frequent and intensive liver function monitoring. As liver function testing alone is insufficient to achieve timely detection of all Drug Induced Liver Injury (DILI) cases, clinicians and participants should be educated to respond appropriately to early symptoms and inform and liaise with study Medical Monitor. Enhanced measures should also be in place for getting liver function test results back rapidly including the recall of participants when liver function tests are significantly elevated.

Should the investigator determine that it is in the best interests of the participant to obtain a specific laboratory result within the shortest time-period possible to safely and appropriately manage the participant, then 2 blood samples must be taken:

1 Send the first sample to the local laboratory (sponsor will reimburse the site for these costs) for immediate processing and reporting and,

2 A duplicate sample central laboratory sample is to be sent via the usual study processes to the designated central study safety laboratory.

Effective referral plans and liaison with medical staff at designated referral hospitals is also essential.

DILI is generally defined as an elevation in liver enzymes that are greater than 3x the upper limit of normal (3xULN) in the context of taking a potentially hepatotoxic drug. Standard anti-TB chemotherapy is known to cause derangement of liver function tests in a substantial number of patients [1]. In many cases this will be asymptomatic and self-limiting. In some cases, severe hepatitis and even fulminant liver failure and death can occur.

In pre-marketing clinical trials of new drugs and regimens, it is especially important to identify and carefully manage any participants who are at risk of progression to serious liver injury. The observation of altered liver function to a degree that has a high risk of progressing to liver failure has been referred to informally as **Hy's Law**; this reflects that pure hepatocellular injury sufficient to cause hyperbilirubinemia is an ominous indicator of the potential for a drug to cause serious liver injury. Briefly, Hy's Law cases have the following three components:

1. The drug causes hepatocellular injury, generally shown by a higher incidence of 3-fold or greater elevations above the ULN of ALT or AST than the (non-hepatotoxic) control drug or placebo.
2. Among participants showing such aminotransferase (AT) elevations, often with ATs much greater than 3x ULN, one or more also show elevation of serum total bilirubin (TBL) to >2x ULN, without initial findings of cholestasis [elevated serum alkaline phosphatase (ALP)].
3. No other reason can be found to explain the combination of increased AT and total bilirubin level (TBL), such as viral hepatitis A, B, or C; pre-existing or acute liver disease; or other drug(s) capable of causing the observed injury.

During the trial, liver function will be monitored regularly with clinical assessment and safety blood tests in participants and this will assist in follow up laboratory measurements that can document either resolution of abnormalities or signal the potential for DILI. In a clinical trial of new drugs and combinations, it is especially important for investigators to follow closely any participants who have evidence of hepatic inflammation or potential toxicity.

2. Patient Enrollment, Site Facilities, and Management Pathways in the Context of Hepatotoxicity in ZeNix

2.1 Considerations When Screening and Enrolling Patients

The investigators should use their own judgement during the screening process for the trial, as well as the Inclusion and Exclusion criteria in the protocol, and carefully consider the patient's background and social circumstances and any influence this may have on the patient's liver function profile during the trial.

Alcohol abuse, nutritional status, other medications (e.g. paracetamol), work- related exposure to hepatotoxic compounds, and traditional/herbal remedies can all have a negative effect on an individual's liver function, and they can act to potentiate the effects of other medication.

If the investigator thinks that a patient may be excessively susceptible to liver injury because of these or other circumstances, then it is reasonable to decide against enrolling the patient into the trial based on this consideration.

Blood tests for liver function will be taken routinely at screening and at the specific time points designated in the protocol, and at Early Withdrawal.

2.2 Education of the Patient during Consent Regarding Liver Dysfunction

During the informed consenting process, it is important that participants are made aware of the risk of liver dysfunction in the trial, the symptoms that may be associated with liver injury, and also how serious this can be if ignored. Participants should be explicitly made aware of the need to seek medical attention/contact the site if they become unwell during any time while participating in the trial. Specific reference should be made to the signs and symptoms associated with hepatotoxicity at every visit (especially gastrointestinal upset) by the investigator, and to reinforce the need to seek medical attention *urgently* should these signs and symptoms occur.

It is essential that the site ensures they have up-to-date contact details for the participant and the participant's next of kin or any significant other (mobile phone numbers and physical home addresses) and that the participant has the 24-hour contact details of the investigator/site staff. Additionally, every effort should be made to:

- Emphasize the need to inform any doctor with whom participants may consult during their involvement in the trial (including their regular doctor/general practitioner) that the site should be contacted as soon as possible and alerted should any medical problem arise. Contact should be made using the contact details documented on the participant card and, **if possible**, prior to initiation of any treatment
- Make participants aware that the trial site is the first place to seek all medical attention, **if possible**.
- As required, relevant information to the participant should be provided based on the site's Standard Operating Procedure (SOP) or equivalent document on on Emergency Medical Management.
- All site staff should be trained, and the training documented, on what this process at each site, has been described as, in the relevant site document.

3. *Considerations Relating to Blood Tests to Investigate Liver Dysfunction at the Site*

Blood tests for liver function will be taken routinely at screening and at the specific time points designated in the protocol, and at Early Withdrawal.

If at any other visit the clinician suspects derangement of liver function, e.g. the participant describes nausea and vomiting, right upper abdominal pain or is jaundiced, blood should be taken for liver function tests and the participant comprehensively assessed for evidence of hepatitis or hepatic impairment and any potentially contributing causes. The safety laboratory blood tests in the ZeNix trial will be sent to the central laboratory for processing and LFT results should be available to the site **within 48 – 72 hours**.

Should the investigator determine that it is in the best interests of the participant to obtain a specific laboratory result within the shortest time-period possible to safely and appropriately manage the participant, then 2 blood samples must be taken:

1. Send the first sample to the local laboratory (sponsor will reimburse the site for these costs) for immediate processing and reporting and,
2. A duplicate sample is to be sent via the usual study processes to the designated central study safety laboratory.

4. *Background Relating to the Detection and Management of Elevated Liver Enzymes During Treatment in ZeNix*

Although studies have suggested that <u>asymptomatic</u> transaminase elevations occur in 20% of patients treated with standard anti-TB regimens [2,3,4], it is always important to consider liver dysfunction as the underlying pathology in participants who present with acute illness while taking TB treatment in the ZeNix trial. While the text below provides more details on the assessment and management of liver dysfunction, there are several key points to be emphasized:

- Gastrointestinal symptoms including nausea, vomiting, reduced appetite, and diarrhoea must always be taken seriously as they can be the first signal of acute liver decompensation and should prompt the Investigator to send urgent liver function tests to the central laboratory **when they first become aware of these symptoms**
- Review of other hepatotoxic drugs must be carried out and any potential "culprit" drugs withdrawn
- If there is evidence of severe disease (e.g. jaundice, haemodynamic instability, severe gastrointestinal symptoms etc.), then hospital admission should be arranged **without waiting for blood tests to become available**
- Clear instructions as outlined in this document regarding the importance of withholding trial medication in severe liver dysfunction must be provided directly to the participant and the physician managing the participant in hospital.

A thorough history should be taken and physical examination of any participants presenting with liver dysfunction, with particular emphasis on asking about other medications that could cause/worsen liver dysfunction (including anti-retroviral therapy), alcohol and drug use, travel history and any traditional remedies the participant may have taken (see appendix 3 "Risk Factors for Hepatotoxicity"). The management plan for the participant is the Investigator's responsibility and the Medical Monitor is available to offer assistance. A list of medications that we would recommend avoiding in the context of liver dysfunction is provided in Appendix 2.

5. Management of Participants Presenting With Symptoms Suggestive of Liver Dysfunction

This will include any participant presenting to the site with symptoms suggestive of liver dysfunction including, but not limited to: gastrointestinal upset (nausea, vomiting, or diarrhea), abdominal pain, itching, unexplained fatigue or jaundice.

If a participant presents or contacts the site/investigator telephonically, with any of these symptoms, either as part of a scheduled visit or if the participant self-presents because of these symptoms, they must have their liver function tests performed **at the time of their review/presentation and all trial medications stopped immediately**. If the Investigator judges the participant to be clinically unwell, then blood tests should be sent to the central laboratory and processed urgently. If there is uncertainty about whether liver toxicity is present or not it is safer to withhold treatment for a few days whilst this diagnosis is being confirmed.

Should the site / investigator receive information (e.g. via a telephonic call to participant) on participants having symptoms suggestive of liver dysfunction, the participant should be instructed **to stop all trial medication** (or relevant concomitant medication e.g. ARVs) immediately and **come to the site as soon as possible**.

6. Management of Participants with Elevated Liver Enzymes on Routine Blood Tests

Elevated liver enzymes will sometimes be detected at scheduled visits as per the trial protocol, with or without symptoms. The table below summarizes the minimum actions by the site Investigators for

an asymptomatic patient with elevated liver enzymes detected on blood tests taken as part of the trial protocol after the participant has started taking trial medication. A flow chart outlining the overall process of assessment and action with trial medication is also included in Appendix 1.

LFT RESULTS	ACTION REQUIRED
ALT and/or AST elevated outside normal range but<br><br><3xULN†<br><br>Bilirubin normal	- Site to make contact with participant **within 48 hours** and establish if there are any symptoms of liver dysfunction and review other medications<br><br>- Participant to be brought back for clinical review which should also include the assessment of concomitant medications, alcohol, over the counter meds and herbal remedies<br><br>- Consider repeating LFTs* **within 72 hours**<br><br>- Trial medication may be continued if Investigator judges it to be safe
ALT and/or AST elevated to ≥3xULN but <5xULN<br><br>Bilirubin normal	- Site must make contact with participant **within 24 hours** and establish if there are any symptoms of liver dysfunction<br><br>- Participant to be brought back for clinical review which should also include the assessment of concomitant medications, alcohol, over the counter meds and herbal remedies AND repeat LFTs* **within 48 hours** if there are no symptoms, but **within 24 hours** of contact if symptoms are reported (including nausea)<br><br>- Withhold all trial medications while waiting for repeat LFTs<br><br>- Trial Medical Monitor to be informed of participant condition, LFT results, and management plan urgently

ALT and/or<br>AST<br>≥5xULN Bilirubin normal<br>**OR**<br>Bilirubin > X2 ULN regardless of ALT/AST<br>result	- Site must make contact with participant **within 24 hours** and clinically assess the participant on the same day if there are any<br>symptoms of liver dysfunction reported (including nausea)<br>- If there are no symptoms, the participant must be assessed AND blood tests including INR, PT and albumin repeated* within 24 hours of making contact<br>- Participant must be told to **stop taking trial medication** as soon as<br>they are contacted, and trial medication must be withheld until repeat LFT results known<br>- Site doctors must alert trial Medical Monitor of the elevated LFTs and their plan of action of becoming aware of the blood tests

* ALT/AST/Bilirubin/ALP

† If ALT/AST are elevated at >1xULN & <3xULN prior to starting trial medication, then this applies in the context of enzyme results that further elevate after starting trial medication

If the liver enzymes are ≥3xULN (with or without an elevated bilirubin) then the Investigator should have a low threshold for arranging admission to a local hospital, especially if the participant is experiencing gastrointestinal symptoms such as nausea or vomiting. In cases when a participant has liver enzymes elevated to ≥5xULN and symptoms suggesting severe liver dysfunction, then we would strongly recommend arranging for hospital-level monitoring and care.

Investigator can request additional tests from the central lab, in addition to the repeat LFT [e.g. Gamma Glutamyl Transferase (GGT), screening for Hepatitis A, B, C; in order to help rule out other causes of a dysfunctional liver test (e.g. alcohol induced hepatic cell injury, hepatobiliary disease, hepatic viral infection)].

The Medical Monitor/TB Alliance Study Physician is available to assist and advise the site investigators, and if there are concerns on the part of the investigator or they are encountering barriers to effective care for any participant in the trial then please contact the Medical Monitor/TB Alliance Study Physician by email or phone immediately.

7. *Management (or Guidance) Plan of re-introduction of Study Medication*

Liver function tests that are improving should be repeated regularly i.e. every 3 days for the first week then once a week until they return to near baseline values for the participant. Manage the participant symptomatically as required using medications that are not potentially hepatotoxic. Infection control issues must be carefully managed whilst TB medications are being withheld, especially if the participant is still culture positive for acid fast bacilli.

If medication has been temporarily stopped, once the liver function values return to near baseline values*, a decision must be made about further TB management based on the patient's clinical status and medical judgement of the investigator.* The Medical Monitor should be notified within 24 hrs of re-starting treatment. In all cases treatment should be recommenced under close supervision for any evidence of recurrent liver

function abnormalities. Within the first week of reintroduction, liver function tests should be repeated regularly, every 3 days for the first week then once a week thereafter.

If there is a further significant elevation of hepatic enzymes or bilirubin or symptoms of clinical concern after resumption of study medication, the study medication should be withdrawn permanently based on clinical judgement of the investigator. Participants who permanently discontinue study medication should be managed as clinically indicated according to local National TB Programme guidelines. The Medical Monitor/TB Alliance Study Physician can be contacted for further advice when referring to the National Treatment Program (e.g. can help with ensuring expedited provision of central Myco lab test results on last positive MTB isolate i.e.:

- Minimum Inhibitory Concentration against bedaquiline, pretomanid and linezolid
- Drug Susceptibility Testing in liquid culture for streptomycin, rifampicin, isoniazid, ethambutol, moxifloxacin and pyrazinamide, and second line TB drugs including but not limited to fluoroquinolones, and injectables;
- Genotyping of MTB isolate.

The Medical Monitor/TB Alliance Study Physician is available to assist the investigators in both the management of liver toxicity and decisions regarding the holding or re-introduction of trial medication. Investigators must involve the Medical Monitor/TB Alliance Physician in any decisions regarding medication hold or re-start, and there should always be a low threshold for contacting the Medical Monitor/TB Alliance Study Physician in cases of elevated liver enzymes.

8. Considerations for the use of Adverse Event terms when reporting liver dysfunction events in the eCRF

In order to ensure consistent data recording in the trial database, it is recommended that the term “Hepatotoxicity” only be used when raised transaminases are accompanied by either clinical symptoms or when a clinically significant elevation of bilirubin is observed. If the intention is to report elevated transaminases, it is recommended the associated parameters (e.g. “elevated AST”, “elevated ALT” “Hepatic enzymes abnormal”, “Hepatic enzymes increased”, “Transaminases abnormal”, “Transaminases increased”, “LFTs Increased”, “LFTs abnormal”, “Hepatic function abnormal”, “ALT abnormal”, “AST abnormal”, “ALT elevated”, “AST elevated” etc..) be considered as the reporting term.

Appendix 1: Flowchart for Management and Assessment of Liver Dysfunction

** If bilirubin is > X2 ULN, manage as per the pathway for **ALT/AST ≥5xULN**, regardless of ALT or AST result*

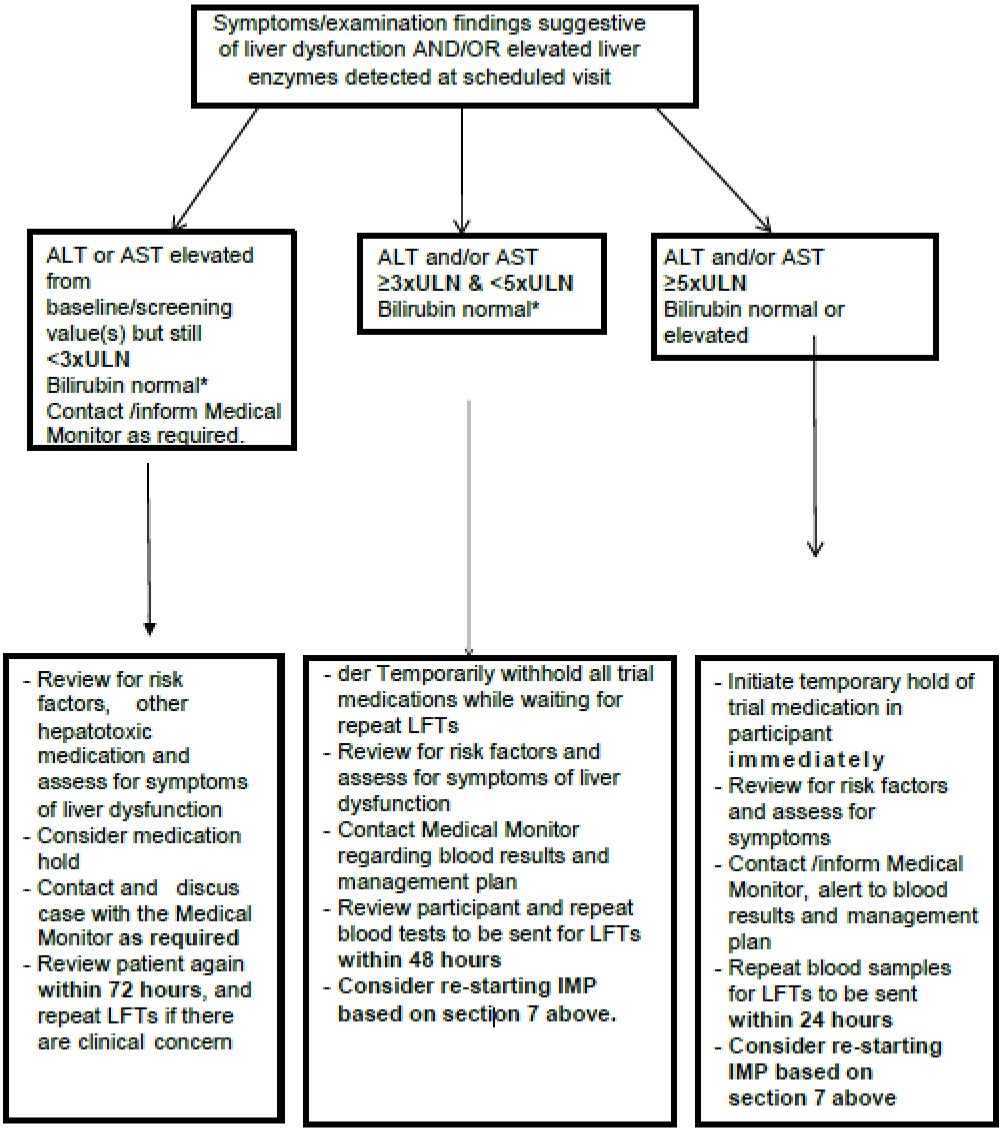

If repeat LFTs return to baseline/near baseline levels, consider re-start of trial medication based on section 8.

Appendix 2: Sample List of Medications to Avoid When Liver Enzymes are Elevated

- Acetaminophen / Paracetamol
- Acetazolamide
- Allopurinol
- Amiodarone
- Amitriptyline
- Amoxicillin
- Amprenavir
- Anti-Retroviral Therapy (as determined by clinical judgement of investigator)
- Atorvastatin
- Augmentin / Co-amoxiclav
- Azathioprine
- Baclofen
- Bumetanide
- Captopril
- Carbamazepine
- Celecoxib
- Chlorpromazine
- Chlorpromazine
- Clindamycin
- Clopidogrel
- Contraceptive pill
- Co-trimoxazole
- Darunavir
- Delavirdine
- Diclofenac
- Doxycycline
- Enalapril
- Fluconazole
- Fluoxetine
- Fosamprenavir
- Furosemide
- Gliclazide
- Glimeperide
- Glipizide
- Ibuprofen
- Irbesartan
- Ketoconazole
- Lisinopril
- Loperamide
- Losartan
- Methotrexate
- Metolazone
- Mirtazepine
- Nitrofurantoin
- Omeprazole
- Other non-steroidal anti-inflammatory drugs
- Paracetamol
- Paroxetine
- Phenobarbital
- Phenothiazines
- Phenytoin
- Pravastatin
- Probenecid
- Prochlorperazine
- Risperidone
- Rosuvastatin
- Sertraline
- Simeprevir
- Simvastatin
- Sodium valproate
- Sotalol
- Sulfasalazine
- Sumatriptan
- Tamsulosin
- Terbinafine
- Tetracycline
- Theophyllin / Uniphyllin
- Tipranavir
- Tolazamide
- Tolbutamide
- Topiramate
- Trazodone
- Tricyclic antidepressants
- Trimethoprim
- Verapamil

Appendix 3: Risk Factors of Hepatotoxicity [5,6]

- Chronic ethanol consumption (alcohol intake of >2 drinks per day (>14 units/ week) in women and >3 drinks per day (>21 units/week) in men is considered the upper threshold for alcohol intake to be considered a risk factor);

- Locally brewed or distilled drinks
- Use of herbal preparations
- Hepatitis (A, B, C and E, cytomegalovirus, and Epstein– Barr virus)
- Pre-existing liver disease or metabolic syndrome (obesity, insulin resistance, diabetes, dyslipidemia)
- Pregnant/3 months post-partum;
- Other hepatotoxic medications;
- Chronic medical conditions (HIV-infection, existing ischemia, severe hypoxemia or congestive heart failure, hypertension).

References

1. FDA Guidance from June 2009 on DILI: Premarketing Clinical Evaluation. 2009. Drug Safety.
2. Shang P, Xia Y, Liu F, Wang X, Yuan Y, Hu D, et al. (2011) Incidence, Clinical Features and Impact on Anti-Tuberculosis Treatment of Anti-Tuberculosis Drug Induced Liver Injury (ATLI) in China. PLoS ONE 6(7): e21836. https://doi.org/10.1371/journal.pone.0021836
3. Sharifzadeh M, Rasoulinejad M, Valipour F, Nouraie M, Vaziri S (2005) Evaluation of patient-related factors associated with causality, preventability, predictability and severity of hepatotoxicity during antituberculosis [correction of antituberclosis] treatment. Pharmacol Res 51: 353–358.M. SharifzadehM. RasoulinejadF. ValipourM. NouraieS. Vaziri2005Evaluation of patient-related factors associated with causality, preventability, predictability and severity of hepatotoxicity during antituberculosis [correction of antituberclosis] treatment.Pharmacol Res51353358
4. Ormerod LP, Horsfield N (1996) Frequency and type of reactions to antituberculosis drugs: observations in routine treatment. Tuber Lung Dis 77: 37–42.LP OrmerodN. Horsfield1996Frequency and type of reactions to antituberculosis drugs: observations in routine treatment.Tuber Lung Dis773742
5. Case Definition and Phenotype Standardization in Drug-Induced Liver Injury *in* Clinical Pharmacology & Therapeutics 89(6):806-15 · June 2011. Available at: https://www.researchgate.net/publication/51099757_Case_Definition_and_Phenotype_Standardization_in_Drug-Induced_Liver_Injury?enrichId=rgreq-0d99f9b3310146b23447a0e250eeef30-XXX&enrichSource=Y292ZXJQYWdlOzUxMDk5NzU3O0FTOjE1NTA1MjQ0MjM5NDYyNEAxNDEzOTc4NzkxNTY5&el=1_x_3&_esc=publicationCoverPdf
6. An official ATS statement: hepatotoxicity of antituberculosis therapy. Saukkonen JJ, Cohn DL, Jasmer RM, Schenker S, Jereb JA, Nolan CM, Peloquin CA, Gordin FM, Nunes D, Strader DB, Bernardo J, Venkataramanan R, Sterling TR; ATS (American Thoracic Society) Hepatotoxicity of Antituberculosis Therapy Subcommittee. Am J Respir Crit Care Med. 2006 Oct 15;174(8):935-52. Available at: https://www.thoracic.org/statements/tuberculosis-pneumonia.php

SimpliciTB

Protocol Section 8.3.1 ALT, AST and Bilirubin Elevations

The Investigator should refer to the Hepatoxicity Management Guideline, to appropriately monitor significant elevations of AST, ALT or Bilirubin. A separate Hepatoxicity Management Guideline document has been developed to aid investigators in the management of participants.

When managing participants with elevated liver enzymes at an Unscheduled visit, the Investigator should request additional tests, in addition to the repeat LFT [e.g. Gamma Glutamyl Transferase (GGT), screening for Hepatitis A, B, C; to assist in ruling out other causes of a dysfunctional liver test (e.g. alcohol induced hepatic cell injury, hepatobiliary disease, hepatic viral infection)].

Hepatotoxicity Management Guideline

1. Introduction

This document is intended to act as a guide for investigators during the management of participants in the SimpliciTB (NC008) trial with either suspected or biochemically confirmed liver function test abnormalities.

In this document, we intend to provide guidance to the investigators on the selection of participants for enrolment and the management of liver enzyme elevations detected during the course of the trial. **The investigator is responsible for the management of the participant, and this document should not overrule an investigator's clinical judgement but rather support them in their investigation and treatment of the participant.** The study Medical Monitor should be informed as soon as possible if liver toxicity is suspected or confirmed in a participant.

Previous studies have demonstrated the need for frequent and intensive liver function monitoring. As liver function testing alone is insufficient to achieve timely detection of all Drug Induced Liver Injury (DILI) cases, clinicians and participants should be educated to respond appropriately to early symptoms and inform and liaise with study Medical Monitor. Enhanced measures should also be in place for getting liver function test results back rapidly, including the recall of participants when liver function tests are significantly elevated.

Should the investigator determine that it is in the best interests of the participant to obtain a specific laboratory result within the shortest time-period possible to safely and appropriately manage the participant, then 2 blood samples must be taken:

1 Send the first sample to the local laboratory (sponsor will reimburse the site for these costs) for immediate processing and reporting and,

2 A duplicate sample is to be sent via the usual study processes to the designated central study safety laboratory.

Effective referral plans and liaison with medical staff at designated referral hospitals is essential.

DILI is generally defined as an elevation in liver enzymes that are greater than 3x the upper limit of normal (3xULN), in the context of taking a potentially hepatotoxic drug. Standard anti-TB chemotherapy is known to cause derangement of liver function tests in a substantial number of participants [1]. In many cases this will be asymptomatic and self-limiting. In some cases, severe hepatitis and even fulminant liver failure and death can occur.

In pre-marketing clinical trials of new drugs and regimens, it is especially important to identify and carefully manage any participants who are at risk of progression to serious liver injury. The observation of altered liver function to a degree that has a high risk of progressing to liver failure has been referred to informally as **Hy's Law**; this reflects that pure hepatocellular injury sufficient to cause hyperbilirubinemia is an ominous indicator of the potential for a drug to cause serious liver injury. Briefly, Hy's Law cases have the following three components:

1. The drug causes hepatocellular injury, generally shown by a higher incidence of 3-fold or greater elevations above the ULN of ALT or AST than the (non-hepatotoxic) control drug or placebo.

2. Among participants showing such aminotransferase (AT) elevations, often with ATs much greater than 3x ULN, one or more also show elevation of serum total bilirubin (TBL) to >2x ULN, without initial findings of cholestasis [elevated serum alkaline phosphatase (ALP)].
3. No other reason can be found to explain the combination of increased AT and total bilirubin level (TBL), such as viral hepatitis A, B, or C; pre-existing or acute liver disease; or other drug(s) capable of causing the observed injury.

During the trial, liver function will be monitored regularly with clinical assessment and safety blood tests in participants and this will assist in follow-up laboratory measurements that can document either resolution of abnormalities or signal the potential for DILI. In a clinical trial of new drugs and combinations, it is especially important for investigators to follow closely any participants who have evidence of hepatic inflammation or potential toxicity.

2. *Participant Enrollment, Site Facilities, and Management Pathways in the Context of Hepatotoxicity in SimpliciTB*

2.1 Considerations When Screening and Enrolling Participants

The investigators should use their own judgement during the screening process for the trial, as well as the Inclusion and Exclusion criteria in the protocol, and carefully consider the participant's background and social circumstances and any influence this may have on the participant's liver function profile during the trial.

Alcohol abuse, nutritional status, other medications (e.g. paracetamol), work-related exposure to hepatotoxic compounds, and traditional/herbal remedies can all have a negative effect on an individual's liver function, and they can act to potentiate the effects of other medication.

If the investigator thinks that a participant may be excessively susceptible to liver injury because of these or other circumstances, then it is reasonable to decide against enrolling the participant into the trial based on this consideration.

Blood tests for liver function will be taken routinely at screening and at the specific time points designated in the protocol, and at Early Withdrawal.

2.2 Education of the Participant during Consent Regarding Liver Dysfunction

During the informed consenting process, it is important that participants are made aware of the risk of liver dysfunction in the trial, the symptoms that may be associated with liver injury, and also how serious this can be if ignored. Participants should be explicitly made aware of the need to seek medical attention/contact the site if they become unwell, during any time while participating in the trial. Specific reference should be made to the signs and symptoms associated with hepatotoxicity at every visit (especially gastrointestinal upset) by the investigator, and to reinforce the need to seek medical attention *urgently* should these signs and symptoms occur.

It is essential that the site ensures they have up-to-date contact details for the participant and the participant's next of kin or any significant other (mobile phone numbers and physical home addresses), and that the participant has the 24-hour contact details of the investigator / site staff. Additionally, every effort should be made to:

- Emphasize the need to inform any doctor with whom participants may consult during their involvement in the trial (including their regular doctor / general practitioner) that the site should be contacted as soon as possible and alerted should any medical problem arise. Contact should be made

using the contact details documented on the participant card and, **if possible**, prior to initiation of any treatment.

- Make participants aware that the trial site is the first place to seek all medical attention, **if possible**.
- As required, relevant information to the participant should be provided based on the site's Standard Operating Procedure (SOP) or equivalent document on 'Emergency Medical Management'.
- All site staff should be trained, and the training documented, on what this process at each site, has been described as, in the relevant site document.

3. *Considerations Relating to Blood Tests to Investigate Liver Dysfunction at the Site*

Blood tests for liver function will be taken routinely at screening and at the specific time points designated in the protocol, and at Early Withdrawal.

If at any other visit the clinician suspects derangement of liver function, e.g. the participant describes nausea and vomiting, right upper abdominal pain or is jaundiced, blood should be taken for liver function tests and the participant comprehensively assessed for evidence of hepatitis or hepatic impairment and any potentially contributing causes. The safety laboratory blood tests in the SimpliciTB trial will be sent to the central laboratory for processing and LFT results should be available to the site **within 48 – 72 hours**.

Should the investigator determine that it is in the best interests of the participant to obtain a specific laboratory result within the shortest time-period possible to safely and appropriately manage the participant, then 2 blood samples must be taken:

1. Send the first sample to the local laboratory (sponsor will reimburse the site for these costs) for immediate processing and reporting and,
2. A duplicate sample is to be sent via the usual study processes to the designated central study safety laboratory.

4. *Background Relating to the Detection and Management of Elevated Liver Enzymes During Treatment in SimpliciTB*

Although studies have suggested that asymptomatic transaminase elevations occur in 20% of participants treated with standard anti-TB regimens [2,3,4], it is always important to consider liver dysfunction as the underlying pathology in participants who present with acute illness while taking TB treatment in the SimpliciTB trial. While the text below provides more details on the assessment and management of liver dysfunction, there are several key points to be emphasized:

- Gastrointestinal symptoms including nausea, vomiting, reduced appetite, and diarrhoea must always be taken seriously as they can be the first signal of acute liver decompensation and should prompt the Investigator to send urgent liver function tests to the central laboratory **when they first become aware of these symptoms**
- Review of other hepatotoxic drugs must be carried out and any potential "culprit" drugs withdrawn
- If there is evidence of severe disease (e.g. jaundice, haemodynamic instability, severe gastrointestinal symptoms etc.), then hospital admission should be arranged **without waiting for blood tests to become available**

- Clear instructions as outlined in this document regarding the importance of withholding trial medication in severe liver dysfunction must be provided directly to the participant and the physician managing the participant in hospital.

A thorough history should be taken and physical examination of any participants presenting with liver dysfunction, with particular emphasis on asking about other medications that could cause/worsen liver dysfunction (including anti-retroviral therapy), alcohol and drug use, travel history and any traditional remedies the participant may have taken (see appendix 3 “Risk Factors for Hepatotoxicity”). The management plan for the participant is the Investigator’s responsibility and the Medical Monitor is available to offer assistance. A list of medications that we would recommend avoiding in the context of liver dysfunction is provided in Appendix 2.

5. *Management of Participants Presenting with Symptoms Suggestive of Liver Dysfunction*

This will include any participant presenting to the site with symptoms suggestive of liver dysfunction including, but not limited to: gastrointestinal upset (nausea, vomiting, or diarrhea), abdominal pain, itching, unexplained fatigue or jaundice.

If a participant presents or contacts the site / investigator telephonically, with any of these symptoms, either as part of a scheduled visit or if the participant self-presents because of these symptoms, they must have their liver function tests performed **at the time of their review/presentation and all trial medications stopped immediately**. If the Investigator judges the participant to be clinically unwell, then blood tests should be sent to the central laboratory and processed urgently. If there is uncertainty about whether liver toxicity is present or not it is safer to withhold treatment for a few days whilst this diagnosis is being confirmed.

Should the site / investigator receive information (e.g. via a telephonic call to participant) on participants having symptoms suggestive of liver dysfunction, the participant should be instructed **to stop all trial medication** (or relevant concomitant medication e.g. ARVs) immediately and **come to the site as soon as possible**.

6. *Management of Participants with Elevated Liver Enzymes on Routine Blood Tests*

Elevated liver enzymes will sometimes be detected at scheduled visits as per the trial protocol, with or without symptoms.

The table below summarizes the minimum actions by the site Investigators for an asymptomatic participant with elevated liver enzymes detected on blood tests taken as part of the trial protocol after the participant has started taking trial medication. A flow chart outlining the overall process of assessment and action with trial medication is also included in Appendix 1.

LFT RESULTS	ACTION REQUIRED

ALT and/or AST elevated outside normal range but<br><br><3xULN†<br><br>Bilirubin normal	- Site to make contact with participant **within 48 hours** and establish if there are any symptoms of liver dysfunction and review other medications<br><br>- Participant to be brought back for clinical review which should also include the assessment of concomitant medications, alcohol, over the counter meds and herbal remedies<br><br>- Consider repeating LFTs* **within 72 hours**<br><br>- Trial medication may be continued if Investigator judges it to be safe
ALT and/or AST elevated to ≥3xULN but <5xULN<br><br>Bilirubin normal	- Site must make contact with participant **within 24 hours** and establish if there are any symptoms of liver dysfunction<br><br>- Participant to be brought back for clinical review which should also include the assessment of concomitant medications, alcohol, over the counter meds and herbal remedies AND repeat LFTs* **within 48 hours** if there are no symptoms, but **within 24 hours** of contact if symptoms are reported (including nausea)<br><br>- Withhold all trial medications while waiting for repeat LFTs<br><br>- Trial Medical Monitor to be informed of participant condition, LFT results, and management plan urgently
ALT and/or AST<br><br>≥5xULN Bilirubin normal<br><br>**OR**<br><br>Bilirubin > X2 ULN regardless of ALT/AST result	- Site must make contact with participant **within 24 hours** and clinically assess the participant on the same day if there are any symptoms of liver dysfunction reported (including nausea)<br><br>- If there are no symptoms, the participant must be assessed AND blood tests including INR, PT and albumin repeated* within 24 hours of making contact<br><br>- Participant must be told to **stop taking trial medication** as soon as they are contacted, and trial medication must be withheld until repeat LFT results known<br><br>- Site doctors must alert trial Medical Monitor of the elevated LFTs and their plan of action of becoming aware of the blood tests

* ALT/AST/Bilirubin/ALP

† If ALT/AST are elevated at >1xULN & <3xULN prior to starting trial medication, then this applies in the context of enzyme results that further elevate after starting trial medication

If the liver enzymes are ≥3xULN (with or without an elevated bilirubin) then the Investigator should have a low threshold for arranging admission to a local hospital, especially if the participant is experiencing gastrointestinal symptoms such as nausea or vomiting. In cases when a participant has liver enzymes elevated

to ≥5xULN and symptoms suggesting severe liver dysfunction, then we would strongly recommend arranging for hospital-level monitoring and care.

Investigator can request additional tests from the central lab, in addition to the repeat LFT [e.g. Gamma Glutamyl Transferase (GGT), screening for Hepatitis A, B, C; in order to help rule out other causes of a dysfunctional liver test (e.g. alcohol induced hepatic cell injury, hepatobiliary disease, hepatic viral infection)].

The Medical Monitor/TB Alliance Study Physician is available to assist and advise the investigators, and if there are concerns on the part of the investigator or they are encountering barriers to effective care for any participant in the trial then please contact the Medical Monitor/TB Alliance Study Physician by email or phone immediately.

7. Management (or Guidance) Plan of re-introduction of Study Medication

Liver function tests that are improving should be repeated regularly i.e. every 3 days for the first week then once a week until they return to near baseline values for the participant. Manage the participant symptomatically as required using medications that are not potentially hepatotoxic. Infection control issues must be carefully managed whilst TB medications are being withheld, especially if the participant is still culture positive for acid fast bacilli.

If medication has been temporarily stopped, once the liver function values return to near baseline values*, a decision must be made about further TB management based on the participant's clinical status and medical judgement of the investigator.* The Medical Monitor should be notified within 24 hrs of re-starting treatment. In all cases treatment should be recommenced under close supervision for any evidence of recurrent liver function abnormalities. Within the first week of reintroduction, liver function tests should be repeated regularly, every 3 days for the first week then once a week thereafter.

If there is a further significant elevation of hepatic enzymes or bilirubin or symptoms of clinical concern after resumption of study medication, the study medication should be withdrawn permanently based on clinical judgement of the investigator. Participants who permanently discontinue study medication should be managed as clinically indicated according to local National TB Programme guidelines. The Medical Monitor/TB Alliance Study Physician can be contacted for further advice when referring to the National Treatment Program (e.g. can help with ensuring expedited provision of central Myco lab test results on last positive MTB isolate i.e.:

- Minimum Inhibitory Concentration against bedaquiline, pretomanid and moxifloxacin
- Drug Susceptibility Testing in liquid culture for streptomycin, rifampicin, isoniazid, ethambutol, moxifloxacin and pyrazinamide, and second line TB drugs including but not limited to fluoroquinolones, and injectables;
- Genotyping of MTB isolate.

The Medical Monitor/TB Alliance Study Physician is available to assist the investigators in both the management of liver toxicity and decisions regarding the holding or re-introduction of trial medication. Investigators must involve the Medical Monitor/TB Alliance Study Physician in any decisions regarding medication hold or re-start, and there should always be a low threshold for contacting the Medical Monitor/TB Alliance Study Physician in cases of elevated liver enzymes.

8. Considerations for the use of Adverse Event terms when reporting liver dysfunction events in the eCRF

In order to ensure consistent data recording in the trial database, it is recommended that the term “Hepatotoxicity” only be used when raised transaminases are accompanied by either clinical symptoms or when a clinically significant elevation of bilirubin is observed. If the intention is to report elevated transaminases, it is recommended the associated parameters (e.g. “elevated AST”, “elevated ALT” “Hepatic enzymes abnormal”, “Hepatic enzymes increased”, “Transaminases abnormal”, “Transaminases increased”, “LFTs Increased”, “LFTs abnormal”, “Hepatic function abnormal”, “ALT abnormal”, “AST abnormal”, “ALT elevated”, “AST elevated” etc..) be considered as the reporting term.

Appendix 1: Flowchart for Management and Assessment of Liver Dysfunction

** If bilirubin is > X2 ULN, manage as per the pathway for **ALT/AST ≥5xULN**, regardless of ALT or AST result*

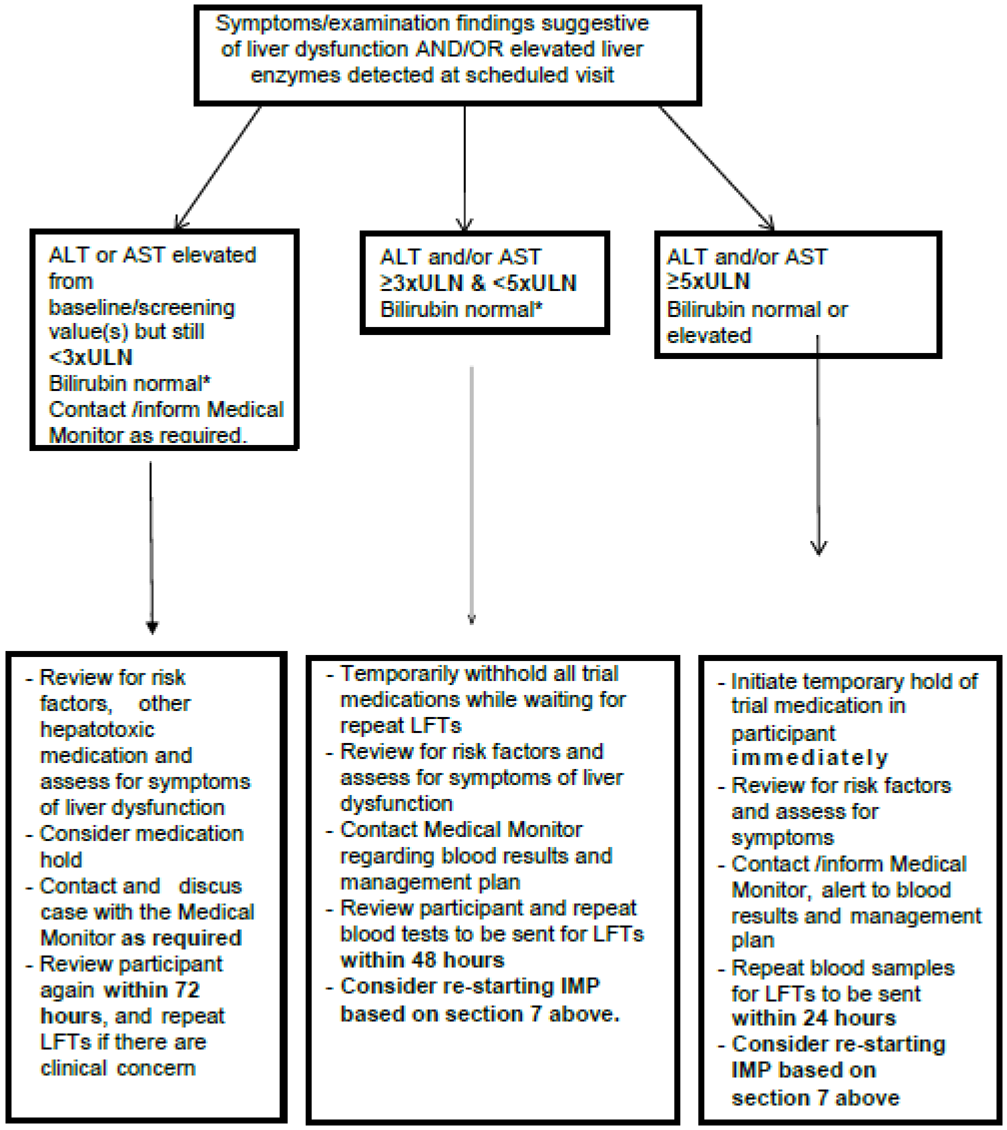

If repeat LFTs return to baseline/near baseline levels, consider re-start of trial medication based on section 8.

Appendix 2: Sample List of Medications to Avoid When Liver Enzymes are Elevated

• Acetaminophen / Paracetamol<br>• Acetazolamide<br>• Allopurinol<br>• Amiodarone<br>• Amitriptyline<br>• Amoxicillin<br>• Amprenavir<br>• Atorvastatin<br>• Augmentin / Co-amoxiclav<br>• Azathioprine<br>• Baclofen<br>• Bumetanide<br>• Captopril<br>• Carbamazepine<br>• Celecoxib<br>• Chlorpromazine<br>• Chlorpromazine<br>• Clindamycin<br>• Clopidogrel<br>• Contraceptive pill<br>• Co-trimoxazole<br>• Darunavir<br>• Delavirdine<br>• Diclofenac<br>• Doxycycline<br>• Enalapril<br>• Fluconazole<br>• Fluoxetine<br>• Fosamprenavir<br>• Furosemide<br>• Gliclazide<br>• Glimeperide<br>• Glipizide<br>• Ibuprofen<br>• Irbesartan<br>• Ketoconazole<br>• Lisinopril	• Loperamide<br>• Losartan<br>• Methotrexate<br>• Metolazone<br>• Mirtazepine<br>• Nitrofurantoin<br>• Omeprazole<br>• Other non-steroidal anti-inflammatory drugs<br>• Paracetamol<br>• Paroxetine<br>• Phenobarbital<br>• Phenothiazines<br>• Phenytoin<br>• Pravastatin<br>• Probenecid<br>• Prochlorperazine<br>• Risperidone<br>• Rosuvastatin<br>• Sertraline<br>• Simeprevir<br>• Simvastatin<br>• Sodium valproate<br>• Sotalol<br>• Sulfasalazine<br>• Sumatriptan<br>• Tamsulosin<br>• Terbinafine<br>• Tetracycline<br>• Theophyllin / Uniphyllin<br>• Tipranavir<br>• Tolazamide<br>• Tolbutamide<br>• Topiramate<br>• Trazodone<br>• Tricyclic antidepressants<br>• Trimethoprim<br>• Verapamil

Appendix 3: Risk Factors of Hepatotoxicity [5,6]

- Chronic ethanol consumption (alcohol intake of >2 drinks per day (>14 units/ week) in women and >3 drinks per day (>21 units/week) in men is considered the upper threshold for alcohol intake to be considered a risk factor);
- Locally brewed or distilled drinks
- Use of local herbal preparations
- Hepatitis (A, B, C and E, cytomegalovirus, and Epstein– Barr virus)
- Pre-existing liver disease or metabolic syndrome (obesity, insulin resistance, diabetes, dyslipidemia)
- Pregnant/3 months post-partum;
- Other hepatotoxic medications;
- Chronic medical conditions (HIV-infection, existing ischemia, severe hypoxemia or congestive heart failure, hypertension).

References

1. FDA Guidance from June 2009 on DILI: Premarketing Clinical Evaluation. 2009. Drug Safety.
2. Shang P, Xia Y, Liu F, Wang X, Yuan Y, Hu D, et al. (2011) Incidence, Clinical Features and Impact on Anti-Tuberculosis Treatment of Anti-Tuberculosis Drug Induced Liver Injury (ATLI) in China. PLoS ONE 6(7): e21836. https://doi.org/10.1371/journal.pone.0021836
3. Sharifzadeh M, Rasoulinejad M, Valipour F, Nouraie M, Vaziri S (2005) Evaluation of participant-related factors associated with causality, preventability, predictability and severity of hepatotoxicity during antituberculosis [correction of antituberclosis] treatment. Pharmacol Res 51: 353–358.M. SharifzadehM. RasoulinejadF. ValipourM. NouraieS. Vaziri2005Evaluation of participant-related factors associated with causality, preventability, predictability and severity of hepatotoxicity during antituberculosis [correction of antituberclosis] treatment.Pharmacol Res51353358
4. Ormerod LP, Horsfield N (1996) Frequency and type of reactions to antituberculosis drugs: observations in routine treatment. Tuber Lung Dis 77: 37–42.LP OrmerodN. Horsfield1996Frequency and type of reactions to antituberculosis drugs: observations in routine treatment.Tuber Lung Dis773742
5. Case Definition and Phenotype Standardization in Drug-Induced Liver Injury *in* Clinical Pharmacology & Therapeutics 89(6):806-15 · June 2011. Available at: https://www.researchgate.net/publication/51099757_Case_Definition_and_Phenotype_Standard ization_in_Drug-Induced_Liver_Injury?enrichId=rgreq-0d99f9b3310146b23447a0e250eeef30-XXX&enrichSource=Y292ZXJQYWdlOzUxMDk5NzU3O0FTOjE1NTA1MjQ0MjM5NDYyNEAxNDEzOT c4NzkxNTY5&el=1_x_3&_esc=publicationCoverPdf

6. An official ATS statement: hepatotoxicity of antituberculosis therapy. Saukkonen JJ, Cohn DL, Jasmer RM, Schenker S, Jereb JA, Nolan CM, Peloquin CA, Gordin FM, Nunes D, Strader DB, Bernardo J, Venkataramanan R, Sterling TR; ATS (American Thoracic Society) Hepatotoxicity of Antituberculosis Therapy Subcommittee. Am J Respir Crit Care Med. 2006 Oct 15;174(8):935-52. Available at: https://www.thoracic.org/statements/tuberculosis-pneumonia.php

DMID toxicity scale 2007 draft

Source: U.S. National Institute of Allergy and Infectious Diseases, DMID, November 2007 (Draft)

ABBREVIATIONS: Abbreviations utilized in the Table:

ULN = Upper Limit of Normal	LLN = Lower Limit of Normal
R_x = Therapy	Req = Required
Mod = Moderate	IV = Intravenous
ADL = Activities of Daily Living	Dec = Decreased

ESTIMATING SEVERITY GRADE

For abnormalities NOT found elsewhere in the Toxicity Tables use the scale below to estimate grade of severity:

Grade	Severity Rating	Definition
**GRADE 1**	Mild	Transient or mild discomfort (< 48 hours); no medical intervention/therapy required.
**GRADE 2**	Moderate	Mild to moderate limitation in activity - some assistance may be needed; no or minimal medical intervention/therapy required.
**GRADE 3**	Severe	Marked limitation in activity, some assistance usually required; medical intervention/therapy required, hospitalizations possible.
**GRADE 4**	Potentially Life-threatening	Extreme limitation in activity, significant assistance required; significant medical intervention/therapy required, hospitalization or hospice care probable.

SERIOUS OR LIFE-THREATENING AEs

ANY clinical event deemed by the clinician to be serious or life-threatening should be considered a grade 4 event. Clinical events considered to be serious or life-threatening include, but are not limited to: seizures, coma, tetany, diabetic ketoacidosis, disseminated intravascular coagulation, diffuse petechiae, paralysis, acute psychosis, severe depression.

COMMENTS REGARDING THE USE OF THESE TABLES

- Standardized and commonly used toxicity tables (Division of AIDS, NCI's Common Toxicity Criteria (CTC), and World Health Organization (WHO)) have been adapted for use by the Division of Microbiology and Infectious Diseases (DMID) and modified to better meet the needs of subjects in DMID trials.
- For parameters not included in the following Toxicity Tables, sites should refer to the "Guide

For Estimating Severity Grade” located above.

- Criteria are generally grouped by body system.
- Some protocols may have additional protocol specific grading criteria, which will supersede the use of these tables for specified criteria.

HEMATOLOGY				
	**Grade 1**	**Grade 2**	**Grade 3**	**Grade 4**
**Hemoglobin**	9.5 - 10.5 gm/dL	8.0 - 9.4gm/dL	6.5 - 7.9 gm/dL	< 6.5 gm/dL
**Absolute Neutrophil Count**	1000-1500/mm^3	750-999/mm^3	500-749/mm^3	<500/mm^3
**Platelets**	75,000-99,999/mm^3	50,000-74,999/mm^3	20,000-49,999/mm^3	<20,000/mm^3
**WBCs**	11,000-13,000/ mm^3	13,000-15,000 /mm^3	15,000-30,000/mm^3	>30,000 or <1,000 /mm^3
**% Polymorphonuclear Leucocytes + Band Cells**	> 80%	90 – 95%	>95%	----------
**Abnormal Fibrinogen**	Low:<br>100-200 mg/dL<br>High:<br>400-600 mg/dL	Low:<br><100 mg/dL<br>High:<br>>600 mg/dL	Low:<br>< 50 mg/dL<br>----------	Fibrinogen associated with gross bleeding or with disseminated coagulation
**Fibrin Split Product**	20-40 mcg/ml	41-50 mcg/ml	51-60 mcg/ml	> 60 mcg/ml
**Prothrombin Time (PT)**	1.01 - 1.25 x ULN	1.26-1.5 x ULN	1.51 -3.0 x ULN	>3 x ULN
**Activated Partial Thromboplastin (APPT)**	1.01 -1.66 x ULN	1.67 - 2.33 x ULN	2.34 - 3 x ULN	> 3 x ULN
**Methemoglobin**	5.0 - 9.9 %	10.0 - 14.9 %	15.0 - 19.9%	> 20.0 %

CHEMISTRIES				
	**Grade 1**	**Grade 2**	**Grade 3**	**Grade 4**
**Hyponatremia**	130-135 mEq/L	123-129 mEq/L	116-122 mEq/L	< 116 mEq/L or abnormal sodium *with* mental status changes or seizures
**Hypernatremia**	146-150 mEq/L	151-157 mEq/L	158-165 mEq/L	> 165 mEq/L or abnormal sodium *with* mental status changes or seizures
**Hypokalemia**	3.0 - 3.4 mEq/L	2.5 - 2.9 mEq/L	2.0 - 2.4 mEq/L or intensive replacement therapy or hospitalization required	< 2.0 mEq/L or abnormal potassium *with* paresis, ileus or life-threatening arrhythmia
**Hyperkalemia**	5.6 - 6.0 mEq/L	6.1 - 6.5 mEq/L	6.6 - 7.0 mEq/l	> 7.0 mEq/L or abnormal potassium *with* life-threatening arrhythmia
**Hypoglycemia**	55-64 mg/dL	40-54 mg/dL	30-39 mg/dL	<30 mg/dL or abnormal glucose *with* mental status changes or coma

**Hyperglycemia (nonfasting and no prior diabetes)**	116 - 160 mg/dL	161- 250 mg/dL	251 - 500 mg/dL	> 500 mg/dL or abnormal glucose *with* ketoacidosis or seizures
**Hypocalcemia (corrected for albumin)**	8.4 - 7.8 mg/dL	7.7 - 7.0 mg/dL	6.9 - 6.1 mg/dL	< 6.1 mg/dL or abnormal calcium *with* life threatening arrhythmia or tetany
**Hypercalcemia (correct for albumin)**	10.6 - 11.5 mg/dL	11.6 - 12.5 mg/dL	12.6 - 13.5 mg/dL	> 13.5 mg/dL or abnormal calcium *with* life threatening arrhythmia
**Hypomagnesemia**	1.4 - 1.2 mEq/L	1.1 - 0.9 mEq/L	0.8 - 0.6 mEq/L	< 0.6 mEq/L or abnormal magnesium *with* life-threatening arrhythmia
**Hypophosphatemia**	2.0 - 2.4 mg/dL	1.5 -1.9 mg/dL or replacement Rx required	1.0 -1.4 mg/dL intensive therapy or hospitalization required	< 1.0 mg/dL or abnormal phosphate *with* life-threatening arrhythmia
**Hyperbilirubinemia (when accompanied by any increase in other liver function test)**	1.1 - <1.25 x ULN	1.25 - <1.5 x ULN	1.5 – 1.75 x ULN	> 1.75 x ULN
**Hyperbilirubinemia (when other liver function are in the normal range)**	1.1 - <1.5 x ULN	1.5 - <2.0 x ULN	2.0 – 3.0 x ULN	> 3.0 x ULN
**BUN**	1.25 - 2.5 x ULN	2.6 - 5 x ULN	5.1 - 10 x ULN	> 10 x ULN
**Hyperuricemia (uric acid)**	7.5 – 10.0 mg/dL	10.1 – 12.0 mg/dL	12.1 – 15.0 mg/dL	>15.0 mg/dL

**CHEMISTRIES**				
	**Grade 1**	**Grade 2**	**Grade 3**	**Grade 4**
**Creatinine**	1.1 - 1.5 x ULN	1.6 - 3.0 x ULN	3.1 - 6 x ULN	> 6 x ULN or dialysis required

**ENZYMES**				
	**Grade 1**	**Grade 2**	**Grade 3**	**Grade 4**
**AST (SGOT)**	1.1 - <2.0 x ULN	2.0 – <3.0 x ULN	3.0 – 8.0 x ULN	> 8 x ULN
**ALT (SGPT)**	1.1 - <2.0 x ULN	2.0 – <3.0 x ULN	3.0 – 8.0 x ULN	> 8 x ULN
**GGT**	1.1 - <2.0 x ULN	2.0 – <3.0 x ULN	3.0 – 8.0 x ULN	> 8 x ULN
**Alkaline Phosphatase**	1.1 - <2.0 x ULN	2.0 – <3.0 x ULN	3.0 – 8.0 x ULN	> 8 x ULN

**Amylase**	1.1 - 1.5 x ULN	1.6 - 2.0 x ULN	2.1 - 5.0 x ULN	> 5.1 x ULN
**Lipase**	1.1 - 1.5 x ULN	1.6 - 2.0 x ULN	2.1 - 5.0 x ULN	> 5.1 x ULN

**URINALYSIS**				
	**Grade 1**	**Grade 2**	**Grade 3**	**Grade 4**
**Proteinuria**	1+ or 200 mg - 1 gm loss/day	2-3+ or 1- 2 gm loss/day	4+ or 2-3.5 gm loss/day	nephrotic syndrome or > 3.5 gm loss/day
**Hematuria**	microscopic only <10 rbc/hpf	gross, no clots >10 rbc/hpf	gross, with or without clots, OR red blood cell casts	obstructive or required transfusion

**CARDIOVASCULAR**				
	**Grade 1**	**Grade 2**	**Grade 3**	**Grade 4**
**Cardiac Rhythm**		asymptomatic, transient signs, no Rx required	recurrent/persistent ; symptomatic Rx required	unstable dysrythmia; hospitalization and treatment required
**Hypertension**	transient increase > 20 mm/Hg; no treatment	recurrent, chronic increase > 20mm/Hg. /treatment required	acute treatment required; outpatient treatment or hospitalization possible	end organ damage or hospitalization required
**Hypotension**	transient orthostatic hypotension with heart rate increased by <20 beat/min or decreased by <10 mm Hg systolic BP, No treatment required	symptoms due to orthostatic hypotension or BP decreased by <20 mm Hg systolic; correctable with oral fluid treatment	requires IV fluids; no hospitalization required	mean arterial pressure <60mm/ Hg or end organ damage or shock; requires hospitalization and vasopressor treatment
**Pericarditis**	minimal effusion	mild/moderate asymptomatic effusion, no treatment	symptomatic effusion; pain; EKG changes	tamponade; pericardiocentesis or surgery required
**Hemorrhage, Blood Loss**	microscopic/occult	mild, no transfusion	gross blood loss; 1-2 units transfused	massive blood loss; > 3 units transfused

**RESPIRATORY**

	Grade 1	Grade 2	Grade 3	Grade 4
**Cough**	Transient - no treatment	persistent cough; treatment responsive	Paroxysmal cough; uncontrolled with treatment	--------------------
**Bronchospasm, Acute**	transient; no treatment; 70% - 80% FEV_1 of peak flow	requires treatment; normalizes with bronchodilator; FEV_1 50% - 70% (of peak flow)	no normalization with bronchodilator;FEV_1 25% - 50% of peak flow; or retractions present	cyanosis: FEV_1 < 25% of peak flow or intubation necessary
**Dyspnea**	dyspnea on exertion	dyspnea with normal activity	dyspnea at rest	dyspnea requiring Oxygen therapy

**GASTROINTESTINAL**				
	**Grade 1**	**Grade 2**	**Grade 3**	**Grade 4**
**Nausea**	mild or transient; maintains reasonable intake	moderate discomfort; intake decreased significantly; some activity limited	no significant intake; requires IV fluids	hospitalization required;
**Vomiting**	1 episode in 24 hours	2-5 episodes in 24 hours	>6 episodes in 24 hours or needing IV fluids	physiologic consequences requiring hospitalization or requiring parenteral nutrition
**Constipation**	requiring stool softener or dietary modification	requiring laxatives	obstipation requiring manual evacuation or enema	obstruction or toxic megacolon
**Diarrhea**	mild or transient; 3- 4 loose stools/day or mild diarrhea last < 1 week	moderate or persistent; 5-7 loose stools/day or diarrhea lasting >1 week	>7 loose stools/day or bloody diarrhea; or orthostatic hypotension or electrolyte imbalance or >2L IV fluids required	hypotensive shock or physiologic consequences requiring hospitalization
**Oral Discomfort/Dysphagia**	mild discomfort; no difficulty swallowing	some limits on eating/drinking	eating/talking very limited; unable to swallow solid foods	unable to drink fluids; requires IV fluids

NEUROLOGICAL				
	**Grade 1**	**Grade 2**	**Grade 3**	**Grade 4**
**Neuro-Cerebellar**	slight incoordination dysdiadochokinesis	intention tremor, dysmetria, slurred speech; nystagmus	locomotor ataxia	incapacitated
**Psychiatric**	mild anxiety or depression	moderate anxiety or depression; therapy required; change in normal routine	severe mood changes requiring therapy; or suicidal ideation; or aggressive ideation	acute psychosis requiring hospitalization; or suicidal gesture/attempt or hallucinations
**Muscle Strength**	subjective weakness no objective symptoms/ signs	mild objective signs/symptoms no decrease in function	objective weakness function limited	paralysis
**Paresthesia (burning, tingling, etc.)**	mild discomfort; no treatment required	moderate discomfort; non-narcotic analgesia required	severe discomfort; or narcotic analgesia required with symptomatic improvement	incapacitating; or not responsive to narcotic analgesia
**Neuro-sensory**	mild impairment in sensation (decreased sensation, e.g., vibratory, pinprick, hot/cold in great toes) in focal area or symmetrical distribution; or change in taste, smell, vision and/or hearing	moderate impairment (mod decreased sensation, e.g., vibratory, pinprick, hot/cold to ankles) and/or joint position or mild impairment that is not symmetrical	severe impairment (decreased or loss of sensation to knees or wrists) or loss of sensation of at least mod degree in multiple different body areas (i.e., upper and lower extremities)	sensory loss involves limbs and trunk; paralysis; or seizures

MUSCULOSKELETAL				
	**Grade 1**	**Grade 2**	**Grade 3**	**Grade 4**
**Arthralgia (joint pain)**	mild pain not interfering with function	moderate pain, analgesics and/or pain interfering with function but not with activities of daily living	severe pain; pain and/or analgesics interfering with activities of daily living	disabling pain
**Arthritis**	mild pain with inflammation, erythema or joint swelling – but not	moderate pain with inflammation, erythema or joint swelling – interfering	severe pain with inflammation, erythema or joint swelling –and	permanent and/or disabling joint destruction

	interfering with function	with function, but not with activities of daily living	interfering with activities of daily living	
**Myalgia**	myalgia with no limitation of activity	muscle tenderness (at other than injection site) or with moderate impairment of activity	severe muscle tenderness with marked impairment of activity	frank myonecrosis

**SKIN**				
	**Grade 1**	**Grade 2**	**Grade 3**	**Grade 4**
**Mucocutaneous**	erythema; pruritus	diffuse, maculo papular rash, dry desquamation	vesiculation or moist desquamation or ulceration	exfoliative dermatitis, mucous membrane involvement or erythema, multiforme or suspected Stevens-Johnson or necrosis requiring surgery
**Induration**	< 15mm	15-30 mm	>30mm	
**Erythema**	< 15mm	15-30 mm	>30mm	
**Edema**	< 15mm	15-30 mm	>30mm	
**Rash at Injection Site**	< 15mm	15-30 mm	>30mm	
**Pruritus**	slight itching at injection site	moderate itching at injection extremity	itching over entire body	

**SYSTEMIC**				
	**Grade 1**	**Grade 2**	**Grade 3**	**Grade 4**
Allergic Reaction	pruritus without rash	localized urticaria	generalized urticaria; angioedema	anaphylaxis
Headache	mild, no treatment required	transient, moderate; treatment required	severe; responds to initial narcotic therapy	intractable; requires repeated narcotic therapy
Fever: oral	37.7 - 38.5 C or 100.0 - 101.5 F	38.6 - 39.5 C or 101.6 - 102.9 F	39.6 - 40.5 C or 103 - 105 F	> 40 C or > 105 F

Fatigue	normal activity reduced < 48 hours	normal activity decreased 25- 50% > 48 hours	normal activity decreased > 50% can't work	unable to care for self

References

1. Dawson R, Diacon AH, Everitt D, et al. Efficiency and safety of the combination of moxifloxacin, pretomanid (PA-824), and pyrazinamide during the first 8 weeks of antituberculosis treatment: a phase 2b, open-label, partly randomized trial in patients with drug-susceptible or drug-resistant pulmonary tuberculosis. Lancet 2015;385:1738-47 (ClinicalTrials.gov number NCT01498419).
2. Tweed CD, Dawson R, Burger DA, et al. Bedaquiline, moxifloxacin, pretomanid, and pyrazinamide during the first 8 weeks of treatment of patients with drug-susceptible or drug-resistant pulmonary tuberculosis: a multicentre, open-label, partially randomized, phase 2b trial. Lancet Respir Med 2019;7:1048-58 (ClinicalTrials.gov number NCT02193776).
3. Tweed CD, Wills GH, Crook AM, et al. A partially randomized trial of pretomanid, moxifloxacin and pyrazinamide for pulmonary TB. Int J Tuberc Lung Dis 2021;25(4):305-314 (ClinicalTrials.gov number NCT02342886).
4. Conradie F, Diacon AH, Ngubane N, et al. Treatment of highly drug-resistant pulmonary tuberculosis. N Engl J Med 2020; 382: 893-902 (ClinicalTrials.gov number NCT02333799).
5. Conradie F, Bagdasaryan TR, Borisov S, et al. Bedaquiline-Pretomanid-Linezolid regimens for drug-resistant tuberculosis. N Engl J Med 2022;387:810-23 (ClinicalTrials.gov number NCT03086486).
6. Cevik M, Thompson L, Upton C, et al. Efficacy, safety, and tolerability of bedaquiline, pretomanid, moxifloxacin and pyrazinamide for the treatment of drug-sensitive and drug-resistant pulmonary tuberculosis. Submitted (ClinicalTrials.gov number NCT03338621)
7. Salinger DH, Subramoney V, Everitt D, Nedelman JR. Population pharmacokinetics of the antituberculosis agent pretomanid. Antimicrobial Agents and Chemotherapy 2019;63(10):e00907-19.
